# Synergistic Effects in Multistate Black Electro‐Thermochromic Smart Windows for Energy‐Efficient Modulation of Solar Heating

**DOI:** 10.1002/advs.77765

**Published:** 2026-09-23

**Authors:** Xilu Wu, Lorenzo Vallan, Hao Li, Jaume Ramon Otaegui, Silvia Mena, Weizhong Jiang, Hongzhi Wang, Jonathan C. Barnes, Daniel Ruiz‐Molina, Kerui Li, Jordi Hernando, Claudio Roscini, Gonzalo Guirado

**Affiliations:** ^1^ State Key Laboratory of Advanced Fiber Materials College of Materials Science and Engineering Donghua University Shanghai China; ^2^ Catalan Institute of Nanoscience and Nanotechnology (ICN2) CSIC and The Barcelona Institute of Science and Technology (BIST) Barcelona Spain; ^3^ Departament de Química Universitat Autònoma de Barcelona Barcelona Spain; ^4^ Department of Chemistry Washington University in St. Louis St. Louis Missouri USA

**Keywords:** electrochromism, photothermal heating, redox‐matched, smart windows, thermochromism

## Abstract

Electrochromic and thermochromic smart windows are mature technologies that offer complementary strategies for regulating solar transmittance. To enhance their overall performance and mitigate key limitations associated with each approach, we present hybrid electro‐thermochromic (ETC) smart windows. By integrating a black electrochromic layer composed of three complementarily‐colored, redox‐matched viologen molecules with a paraffin‐particle‐based thermochromic film, these hybrid devices deliver multiple synergistic functionalities: voltage‐controlled, multimodal operation enabling both passive and active solar modulation; photothermal‐assisted activation of the thermochromic layer driven by the strong solar absorption of the colored electrochromic state; efficient regulation of solar heat gain with a ten‐fold reduction in electrical energy consumption for the electrochromic component; and near‐complete suppression of sunlight transmission by simultaneous activation of both layers thanks to their complementary spectral responses. In energy‐saving experiments in hot days, ETC windows achieved a maximum reduction of 8.5°C in solar heat gain relative to clear glass, in agreement with building‐energy simulations predicting up to a 23% decrease in annual energy usage in low‐latitude regions (e.g., Honolulu). Collectively, these results demonstrate the practical energy‐saving potential of ETC smart windows, which offer a versatile and energy‐efficient solution for dynamic solar control across diverse climatic conditions.

## Graphical abstract

Hybrid electro‐thermochromic smart windows are developed for dynamic climate control by integrating a redox‐matched black electrochromic gel with a paraffin‐based thermochromic film. This synergistic combination enables multimodal solar modulation, where photothermal heating from the electrochromic layer triggers thermochromic activation at lower temperatures and enables on‐command solar heating reduction at lower operation costs.

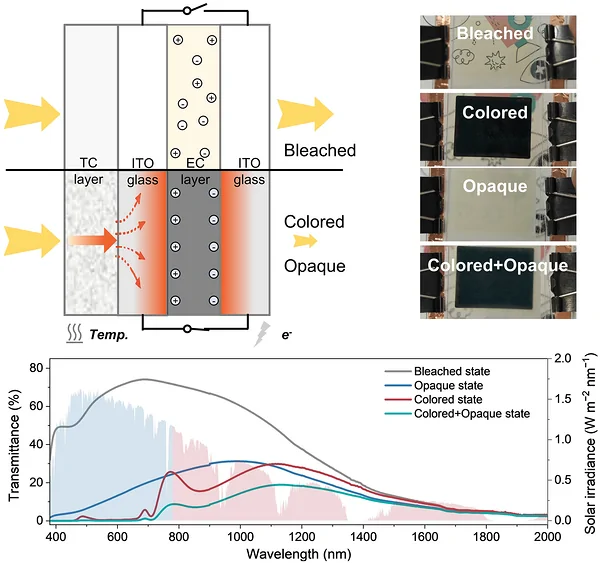

## 1 Introduction

Buildings represent a major contributor to global energy consumption, with heating, ventilation, and air conditioning (HVAC) systems accounting for nearly 20% of residential energy use [1, 2]. A significant portion of this demand arises from light and heat fluxes through windows, a situation aggravated by the widespread adoption of large glazed envelopes in modern construction. Reducing unwanted solar heat gain while maintaining daylight illumination is therefore a key strategy to lower energy demand and improve indoor comfort. Smart windows offer a promising solution toward this goal, dynamically modulating solar radiation transmittance in response to stimuli such as temperature, electric fields or sunlight [3, 4, 5, 6].

Among these, electrochromic (EC) windows allow active, precise, and reversible on‐demand user control of solar radiation transmittance via an applied voltage [7, 8, 9, 10]. Materials such as transition‐metal oxides, conjugated polymers, and viologens undergo redox reactions that alter light absorption, enabling adjustment to user preference. However, their response is frequently confined to a limited spectral range of solar irradiance, as the development of spectrally broad EC windows ―e.g., black EC windows― remains a significant challenge [11, 12, 13, 14, 15]. In addition, since the activation and preservation of the colored state inevitably requires electrical energy, minimizing the operational energy cost is still an important issue to overcome. As an alternative, thermochromic (TC) windows passively regulate solar transmission according to temperature without the need for energy consumption. Most common TC materials, such as vanadium oxides [16, 17, 18], hydrogels [19, 20, 21, 22], ionogels [23, 24, 25] and liquid crystals [26, 27, 28], undergo phase transitions, producing increments in light absorption or scattering once a threshold temperature is reached, thus reducing solar gain without human intervention. Despite this advantage, the fixed switching temperature intrinsic to the phase transition of these materials limits practical use. For instance, a TC window designed to switch at 35°C may remain inactive in cooler seasons, even under strong sunlight. Conversely, a lower activation temperature can trigger the TC response too often, unnecessarily blocking solar heat when needed during mild weather. A partial solution to this issue involves incorporating photothermal agents into TC materials, which also allow them to respond to solar irradiation intensity by converting sunlight into local heat [29, 30, 31, 32, 33, 34, 35, 36]. However, these photothermal agents often reduce transmittance irrespective of weather conditions, even when high light transparency is desired to maximize natural lighting and solar heat gain, while still leaving the user without control over the transition temperature.

To address some of the inherent limitations of EC and TC smart windows, their integration into a single hybrid device has been recently proposed. By independently operating their separate EC and TC components, the resulting electro‐thermochromic (ETC) windows exhibit multimodal behavior, providing both passive and active management of solar energy and user privacy [37, 38, 39, 40, 41, 42]. In addition, a synergistic effect has been recently described for this type of hybrid devices: the electroinduced single‐color blue state of an EC layer was exploited to photothermally assist the active switching of a contiguous TC film, reducing the electricity consumption required to trigger on‐demand thermochromism compared with other approaches, such as Joule‐heating‐induced electrothermochromism [43]. In this work we further refined this concept to fully unleash the potential of hybrid ETC smart windows to offer energy‐efficient modulation of solar irradiation. First, we considered optimization of the photothermally assisted activation of thermochromism in these devices, which requires developing broadband EC layers that do not only absorb in a narrow spectral window, but show maximized sunlight absorption across the visible and near infrared (NIR) regions. As a result, thermochromic responses could be achieved at much lower temperatures. Second, thanks to the additional contribution to sunlight modulation provided by the TC material, we aimed to demonstrate that lower voltages would be required for the EC layer to obtain optimal solar heat reduction and/or privacy levels, ultimately reducing energy consumption and operational cost relative to pure EC smart windows.

**FIGURE 1:**
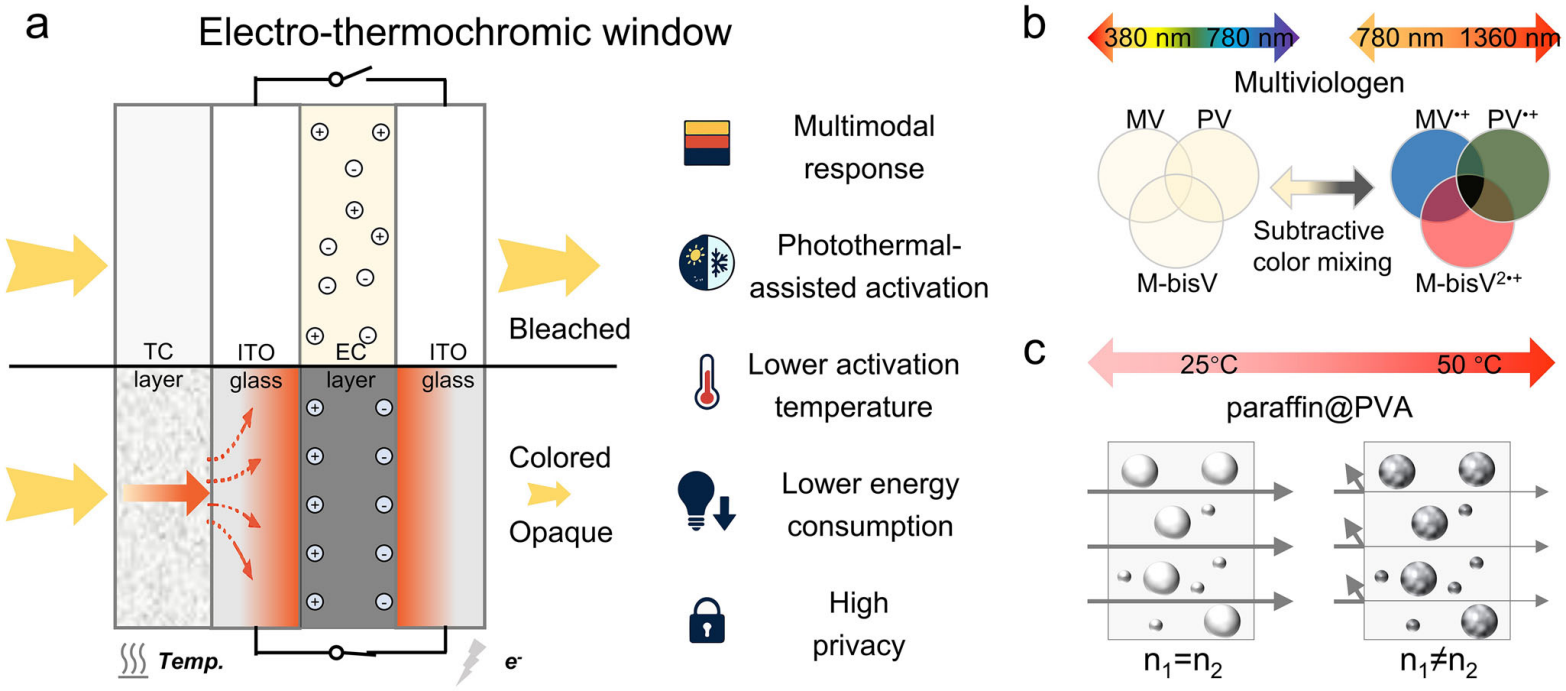
Multistimuli‐responsive smart windows based on combined electrochromic and thermochromic layers. (a) Electro‐thermochromic smart window combining EC and TC layers, in which the photothermally assisted activation strategy enables synchronized optical and thermal modulation for passive cooling, privacy control, and energy savings throughout the year. (b, c) Schematic representation of the operating principles of the EC and TC materials used for the preparation of combined ETC smart windows, which consist of (b) a redox‐matched, triviologen‐loaded electrochromic gel to generate broadband absorption and ultra‐low visible transmittance through a subtractive mixing strategy, and (c) a thermochromic composite film comprising paraffin particles dispersed in a poly(vinyl alcohol) layer (paraffin@PVA), where temperature‐induced paraffin melting leads to refractive index mismatch and increased light scattering.

To this end, herein we pioneered the development of a novel type of electro‐thermochromic windows, where the intertwined operation of their electro‐ and thermochromic units enables optimized solar heating management and energy savings in buildings (Figure 1a). Aiming to maximize the synergistic effects in these hybrid devices, we deliberately selected electrochromic and thermochromic components that differ from those employed in earlier ETC systems. Thus, to enhance photothermal‐assisted activation of the TC component, we fabricated an EC layer with broadband absorption in the electroactivated state, based on multiple viologen molecules designed to meet two principal requirements: (1) complementary absorption to generate near‐black coloration when concomitantly reduced, by means of a subtractive color mixing strategy; and (2) operation at similar reduction potentials, preventing incomplete activation under a fixed applied voltage that leads to unbalanced coloration and reduced long‐term device stability (Figure 1b). As for the thermochromic layer of the hybrid device, we used a TC material recently introduced by us, consisting of a thin poly(vinyl alcohol) (PVA) film embedding paraffin microparticles (Figure 1c) [29, 30, 44]. Paraffin undergoes a solid‐to‐liquid phase transition at its melting temperature (typically, 36°C–37°C for eicosane‐based particles), generating a refractive‐index mismatch in the composite that induces light scattering and reduces transmittance across the visible and near‐infrared regions. Compared to the other TC layers used so far for the fabrication of ETC devices ―hydrogels [38, 39, 40, 41, 43] and vanadium oxide [37] ―, our paraffin‐based material offers several advantages, such as simpler architecture, lower cost and broader, thermally‐tunable responses. In addition, thermoresponsive composites based on paraffins provide further adaptive thermal regulation owing to the latent heat released or absorbed upon phase change, a feature exploited not only in smart windows [45] but also in other functional devices [46, 47]. Consequently, the integration of the paraffin‐based TC layer with the viologen‐based black EC module allows the fabrication of cost‐effective hybrid ETC windows that deliver enhanced, multimode modulation across the visible and NIR solar spectrum, providing a versatile platform for next‐generation smart windows.

## 2 Results and Discussion

### 2.1 Selection of Viologen Molecules for Redox‐Matched, Black Electrochromism

**FIGURE 2:**
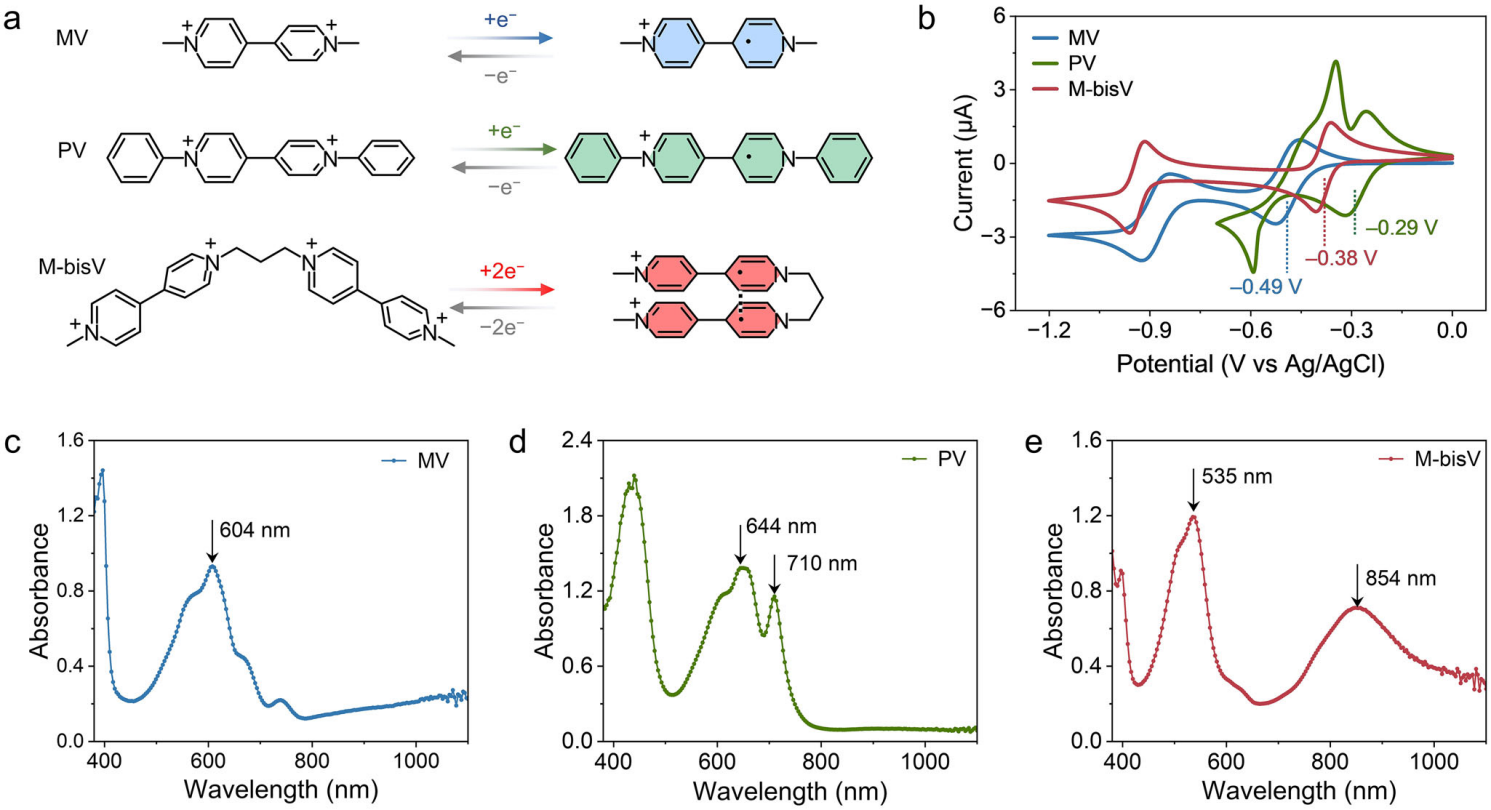
Redox‐matched viologen molecules with subtractive color mixing for near‐black state electrochromism. (a) Electrochromic reactions of MV, PV, and M‐bisV. (b) Cyclic voltammograms of solutions of MV (4 mmol L^−1^), PV (4 mmol L^−1^), and M‐bisV (2 mmol L^−1^) in PC/0.2 mol L^−1^ [EMIM][TFSI] (working electrode: glassy carbon; counter electrode: Pt; reference electrode: Ag/AgCl; scan rate: 0.01 V s^−1^). (c–e) UV–vis absorption spectra of reduced MV^•+^, PV^•+^, and M‐bisV^2•+^ recorded in PC/0.2 mol L^−1^ [EMIM][TFSI] solution (working electrode: Pt mesh; pathlength: 0.2 mm quartz cuvette).

To achieve black electrochromism in our hybrid ETC device through subtractive color mixing, three different viologen electrochromes were employed: methyl viologen (MV) [48], phenyl viologen (PV) [49], and propyl‐bridged bis(methyl viologen) (M‐bisV) [50], which are known to exhibit blue, green, and pink‐red colors upon electrochemical reduction at low potentials, respectively (Figure 2a). As recently reported by us, the choice of M‐bisV also provides a pronounced NIR absorption in the reduced state caused by reversible intramolecular π‐dimer formation (Figure 2a) [50], thereby further broadening the spectral response of our multi‐viologen EC layer and enhancing photothermal conversion to activate the adjacent TC component in the final hybrid device.

The electrochemical behavior of each viologen compound of choice was analyzed via cyclic voltammetry (CV) using a three‐electrode cell in propylene carbonate (PC) containing 1‐ethyl‐3‐methylimidazolium bis(trifluoromethylsulfonyl)imide ([EMIM][TFSI]) as the electrolyte, selected for its wide electrochemical stability window and high ionic conductivity. As shown in Figure 2b, all viologens exhibited two reduction waves within the cathodic potential range scanned. The first of these waves corresponds to the reversible transition between the initial colorless oxidized species (MV, PV and M‐bisV) and the singly reduced radical forms with the desired blue, green or pink‐red color, which requires a one‐ (MV^•+^ and PV^•+^) or two‐electron transfer process (M‐bisV^2•+^) depending on the number of viologen monomers per molecule (Figure 2a). Notably, similar standard potentials (*E* ^0^) were observed for the three compounds analyzed, with *E* ^0^ values of −0.49, −0.38, and −0.29 V (vs Ag/AgCl) for MV, M‐bisV and PV, respectively. In addition, they are sufficiently separated from the second reduction wave leading to further coloration changes and/or viologen degradation [51], even for the most reducible compound considered herein (*E*_pc_ = −0.59 V for PV, where *E*_pc_ corresponds to the cathodic peak potential value). Consequently, MV, PV, and M‐bisV should undergo simultaneous electrochromic activation within a narrow and well‐defined voltage window (only 0.20 V between MV and M‐bisV), enabling synchronized coloration under a fixed applied voltage to reach the target near‐black color. It must be noted that such a condition would not be met by replacing M‐bisV with other, more common red‐absorbing viologens such as monoheptyl viologen (Mono‐HV), which display significantly more negative first reduction potentials [52]. Thus, Mono‐HV exhibits a reduction peak at approximately *E* ^0^ = −1.02 V, which lies well outside the operating voltage window of the redox‐matched triviologen molecules chosen herein (Figure S1a). Incorporating such a viologen molecule would require a higher driving voltage to induce coloration, potentially resulting in over‐reduction or degradation of other viologen molecules (e.g., PV or MV), thereby causing color imbalance, irreversible electrochemical side reactions and coloration, and compromised device stability (Figure S1b).

In addition to redox matching, the three viologen molecules were selected for their complementary optical absorption in the reduced state. UV–vis absorption spectra of MV^•+^, PV^•+^, and M‐bisV^2•+^ were recorded in a three‐electrode cell, with radicals generated in situ using a platinum mesh working electrode. As expected, each radical exhibits complementary absorption in a distinct spectral region, with MV^•+^, PV^•+^, and M‐bisV^2•+^ presenting absorption bands centered at λ_abs, max_ = 604 nm (blue color), across 450–650 nm (green color), and around λ_abs, max_ = 535 nm (pink‐red color), respectively (Figure 2c–e). These complementary absorption characteristics of MV^•+^, PV^•+^ and M‐bisV^2•+^ in terms of intensity, bandwidth, and spectral range enable their reduced forms, when combined, to block nearly the entire visible spectrum, providing a robust spectral basis for achieving a near‐black EC device. Moreover, radical ion paring in M‐bisV^2•+^ allowed to extend the light absorption into the NIR range (λ_abs, max_ = 854 nm), a critical feature to enhance solar heat gain regulation with smart windows.

### 2.2 Electrochemical and Electrochromic Properties Of Viologen‐Based EC Gels and Devices

**FIGURE 3:**
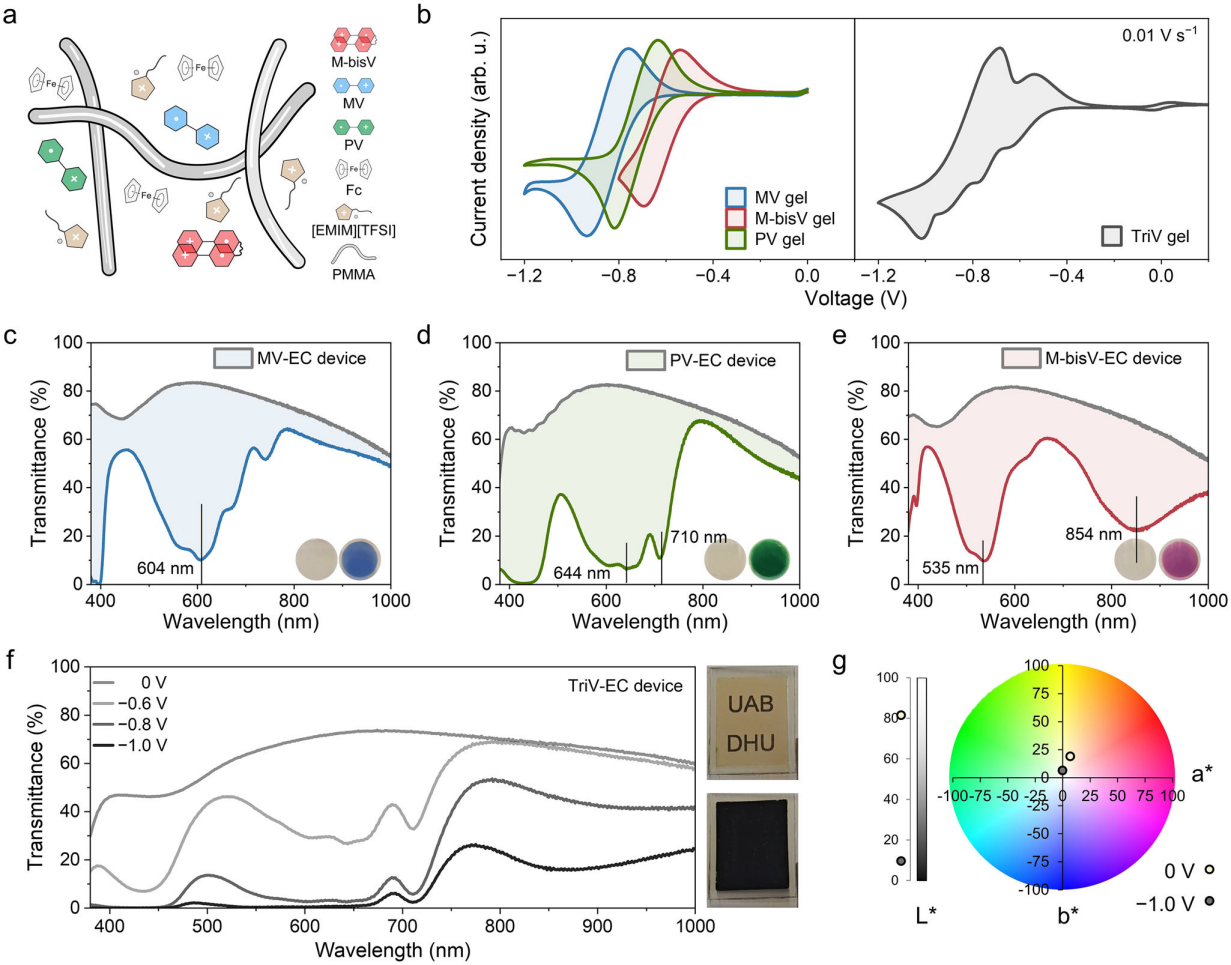
Electrochromic performance of MV‐, PV‐, M‐bisV‐, and TriV‐based EC devices. (a) Schematic illustration of EC gels. (b) Cyclic voltammograms of the viologen‐based EC devices recorded at a scan rate of 0.01 V s^−1^. (c–f) UV–vis transmittance spectra and visual photographs of MV‐, PV‐, M‐bisV‐, and TriV‐based EC devices in the bleached (0 V) and colored state (−1.0 V). (g) CIE 1976 Lab color coordinates of the TriV‐based EC device in the bleached and colored state.

EC gels were developed to enable the fabrication of all‐in‐one EC devices, which were composed of each (MV, PV, M‐bisV) or all (TriV) of the three viologens selected as electrochromes, Fc as an auxiliary redox mediator, poly(methyl methacrylate) (PMMA) as a polymer matrix, PC as the solvent, and [EMIM][TFSI] as an electrolyte (Figure 3a). In these formulations, PMMA forms a robust three‐dimensional polymer network that ensures uniform dispersion of all active components while providing the structural stability required for device assembly. The rheological properties of the TriV‐based EC gel were characterized to evaluate processing suitability and structural stability. At a shear rate of 10 s^−1^, the EC gel showed a viscosity of approximately 6770 mPa s, which is orders of magnitude higher than that of an equivalent liquid EC electrolyte without PMMA, ranging from 2.3 to 5.4 mPa s (Figure S2). This elevated viscosity enhances handling safety, effectively preventing electrolyte leakage and maintaining intimate electrode‐electrolyte interfacial contact during EC device operation. As for the ionic transport of the different viologen‐based EC gels prepared, it was evaluated by electrochemical impedance spectroscopy (EIS) (Figure S3). From this data, the ionic conductivities (σ) determined for the MV‐, PV‐, M‐bisV‐ and TriV‐based EC gels were 4.28×10^−4^, 3.76×10^−4^, 4.09×10^−4^ and 4.22×10^−4^ S cm^−1^, respectively. These values are indicative of rapid ion transport in the gels, enabling efficient charge injection and stable multicolor subtractive electrochromic operation.

For the fabrication of EC devices, all viologen‐based EC gels were sandwiched between two indium tin oxide (ITO) electrodes deposited onto glass (EC gel thickness ∼ 0.02 cm). The electrochemical redox behavior of the resulting devices was first investigated by CV. As shown in Figure 3b, the standard potentials (*E* ^0^) of the first reduction wave for MV‐, PV‐, and M‐bisV‐based gels were determined to be −0.86, −0.64, and −0.72 V, respectively, confirming that all three viologens can be activated within a closely spaced potential window (0.22 V). In fact, these waves were found to largely overlap when measuring the cyclic voltammogram of the TriV‐based EC device containing all viologens (Figure 3b). To evaluate the transport kinetics of viologen molecules within the gel matrices, scan rate‐dependent CV measurements were also performed. As shown in Figure S4 for MV‐, PV‐, and M‐bisV‐based EC devices, the peak current of the first reduction step scaled linearly with the square root of the scan rate (*v* ^1/2^), indicating that the redox processes are predominantly diffusion‐controlled. Using the Randles‐Ševčík equation, the apparent diffusion coefficients (*D*) in these gels were calculated to be 2.36×10^−7^ cm^2^ s^−1^ for MV, 1.97×10^−7^ cm^2^ s^−1^ for PV, and 8.82×10^−8^ cm^2^ s^−1^ for M‐bisV (Table S1). The lower *D* value determined for the M‐bisV‐based EC gel is attributed to the larger molecular size of the viologen dimer consisting of two linked bipyridinium structures, resulting in hindered mobility within the polymeric network. Nevertheless, all three viologen molecules possess sufficient diffusivity to enable rapid switching and excellent cycling stability.

The UV–vis transmittance spectra of the MV‐, PV‐, M‐bisV‐, and TriV‐based EC devices were recorded in both the bleached state at 0 V and the fully colored state at −1.0 V. As shown in Figure 3c–f and Figure S5, the MV‐, PV‐, and M‐bisV‐based devices show transmittance variations of 73.2% at 604 nm, 75.2%/67.4% at 644/710 nm, and 69.8%/44.4% at 535/854 nm, respectively, corresponding to separate blue, green, and pink‐red EC states. In contrast, the TriV‐based EC device, which remained highly transparent at 0 V with an average visible transmittance (AVT) of 65.1%, showed almost complete suppression of transmittance across the entire visible range (380–700 nm) when driven at −1.0 V, producing a near‐black state and additional NIR light attenuation due to M‐bisV electrochromic behavior. Therefore, these results demonstrate the broad modulation range of the TriV‐based EC device, which becomes completely opaque by effectively blocking the entire visible spectrum upon reduction, a very interesting feature for privacy control (Figure 3f). Notably, a similar effect was observed in bendable EC devices fabricated using ITO‐coated poly(ethylene terephthalate) substrates, demonstrating the compatibility of our gel electrolyte with flexible electrochromic configurations. As shown in Figure S6, the EC device prepared exhibited reversible color switching from a transparent to a dark near‐black state under different applied potentials, even when bent, confirming that the gel electrolyte maintains efficient ion transport and interfacial contact within the flexible architecture.

To further quantify the color evolution of each EC device, their CIE 1976 Lab color coordinates were measured in both bleached and colored states. In this color space, a* (red‐green axis) and b* (blue‐to‐yellow axis) represent chromaticity, while L* (black‐white coordinate) indicates lightness. As shown in Figure S7, the MV‐, PV‐, and M‐bisV‐based devices showed high L* values (> 90) and low a* and b* values in the bleached state, confirming excellent initial transparency. Upon viologen reduction at −1.0 V, L* decreased to ca. 55 due to coloration, while the changes observed in a* and b* corresponded to the blue, green, and pink‐red hue developed in each of these devices, respectively. In contrast, the TriV‐based EC device displayed a different electroinduced chromatic variation, with its CIE 1976 Lab color coordinates shifting from [82.35, 2.27, 15.59] at 0 V to [77.42, −8.43, 25.47] at −0.4 V and [46.37, −42.57, 34.33] at −0.6 V, indicating transient greenish tinting at intermediate voltages due to the preferential reduction of the PV^2+^ species at low potential (Table S2 and Figure S8). Upon further increasing the voltage, the device became much darker and increasingly more neutrally colored after concomitant reduction of MV and M‐bisV, with coordinates of [17.45, −22.12, 13.81] and [13.16, 0.33, 3.54] at −0.8 and −1.0 V, respectively (Figure 3g). Notably, a larger luminance drop was observed at ‐1.0 V (ΔL* = –69.19), accompanied by a minimal chroma value in the colored state (*C^∗^* = 3.57 at −1.0 V, see Equation (1) in the Experimental Section), quantitatively confirming the near‐black state achieved and validating the effectiveness of the subtractive color‐mixing strategy applied.

**FIGURE 4:**
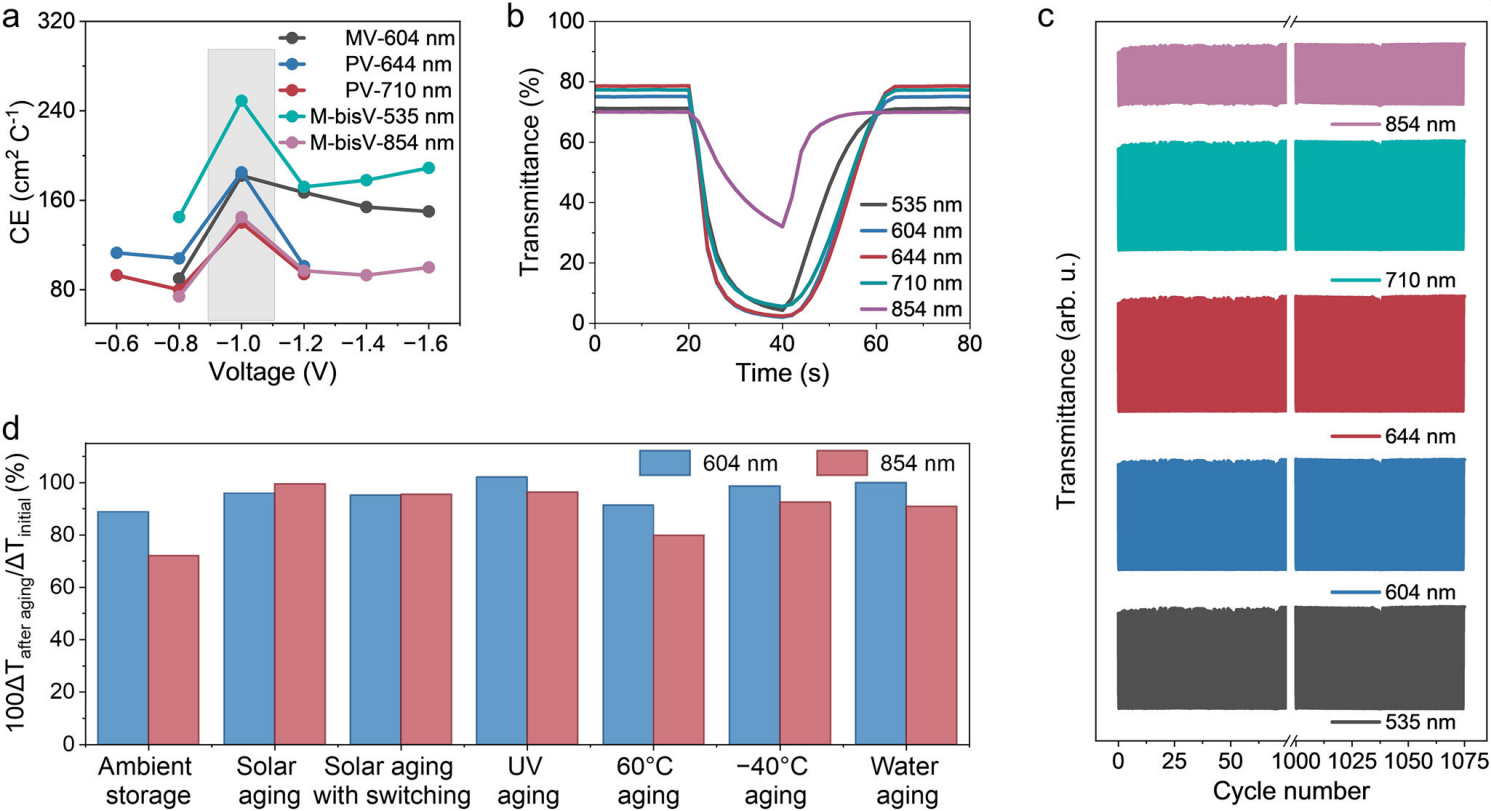
Cycling stability of the TriV‐based EC device enabled by redox‐matching and voltage optimization. (a) Coloring efficiency of MV‐, PV‐, and M‐bisV‐based EC devices under different voltages. (b) Time‐transmittance response curves of the TriV‐based EC device monitored at different wavelengths. (c) Long‐term cyclic test of the TriV‐based EC device at different wavelengths. (d) Preservation of the electrochromic modulation of the TriV‐based EC device at 604 and 854 nm after ambient storage (5 months), aging with a solar simulator (1 sun, 96 h), aging with a solar simulator and periodic electroswitching (1 sun, 96 h, 100 cycles every 12 h), UV aging (133 W m^−2^, 48 h), thermal aging at 60°C (168 h) and −40°C (168 h), and water aging (immersion for 168 h).

We next assessed the coloration efficiency (CE) of our EC devices, which is a key performance metric defined as the change in optical density (ΔOD) per charge density unit (ΔQ). The CE values of each viologen‐based EC device were evaluated at their characteristic absorption wavelengths across a range of applied voltages. All three monoviologen‐based EC devices exhibited a voltage‐dependent CE profile that increased to a maximum around −1.0 V: 182 cm^2^ C^−1^ at 604 nm for the MV‐based EC device; 185 cm C^−1^and 140 cm^2^ C^−1^ at 644 and 710 nm, respectively, for the PV‐based EC device; and 249 cm^2^ C^−1^ and 145 cm^2^ C^−1^ at 535 and 854 nm, respectively, for the M‐bisV‐based EC device (Figure 4a and Figure S9 and Table S3). These CE values fall within the typical range for viologen‐based EC devices [53, 54]. At voltages beyond −1.0 V where charge injection increased, the ΔOD per unit charge decreased leading to lower CE values, probably due to saturation of the optical density in the colored states produced. Based on this balance between optical contrast and efficiency, −1.0 V was selected as the optimal operating voltage for subsequent experiments.

The switching speed of the TriV‐based EC device was evaluated by monitoring its transmittance change during square‐wave voltage steps. This study was conducted at several representative wavelengths that are characteristic of each of the viologens incorporated into the device: 604 nm (for MV), 644 and 710 nm (for PV), and 535 and 854 nm (for M‐bisV). As shown in Figure 4b, when the EC device was cycled between −1.0 V for coloration and 0 V for bleaching, it exhibited rapid and consistent switching across the whole visible and NIR spectrum, indicating similar response times for all viologen electrochromes. In particular, the times required to reach 90% of the full optical contrast ranged from 7.4 s (644 nm) to 15.7 s (854 nm) for coloration, and from 8.8 s (854 nm) to 20.2 s (644 nm) for color bleaching. Overall, both activation and bleaching switching times fell below 20 s, which are considered suitable for practical smart window applications, especially given the broad spectral coverage and substantial optical contrast achieved (Movie S1). Therefore, beyond color complementarity and reduction voltage matching, these viologens also manifest similar activation and bleaching responses, which ensures the preservation of homogeneous neutral coloration all over the switching process.

Furthermore, the long‐term cycling stability of the TriV‐based EC device was evaluated by continuous switching for 1075 cycles, again monitoring transmittance at different wavelengths representative of each viologen electrochrome (535, 604, 644, 710, and 854 nm). As shown in Figure 4c, the EC device maintained stable and repeatable optical modulation throughout 1075 cycles, exhibiting no evident degradation across all wavelengths. Further stability was confirmed by chronoamperometry (CA), which showed nearly identical, symmetric current profiles for the initial and last stages of the cycling experiment, indicating highly reversible charge transfer and negligible degradation of the viologen mixture and gel under prolonged operation (Figure S10a). To further verify the cycling durability, the EC switching test was extended to 11 000 cycles, after which the device still retained almost 90% of its initial transmittance modulation at 604 nm, confirming the robust reversibility of the redox‐matched TriV‐based EC system under prolonged operation (Figure S10b).

Finally, we investigated the aging stability of the TriV‐based EC device under different environmental and operational conditions. As summarized in Figure 4d and Figure S11, the variation of EC transmittance modulation in the visible (ΔT_vis_) and NIR (ΔT_NIR_) spectral ranges was evaluated for the following aging tests: (1) ambient storage for 5 months, after which the device retained about 90% and 75% of the initial ΔT_vis_ and ΔT_NIR_ values, respectively; (2) irradiation with a solar simulator (AM1.5G, 1000 W m^−2^) for 96 h, with or without simultaneous EC switching (100 cycles every 12 h), which resulted in negligible losses in transmittance modulation (< 5%); (3) UV irradiation (365 nm, 133 W m^−2^) for 48 h, which did not deteriorate the EC performance either (< 5% changes in ΔT_vis_ and ΔT_NIR_); (4) exposure to high temperatures (60°C) for 168 h, leading to modest decrements in ΔT_vis_ (∼9%) and ΔT_NIR_ (∼20%); (5) exposure to low temperatures (−40°C) for 168 h, which had an even smaller impact on EC switching amplitude (< 7.5% changes in ΔT_vis_ and ΔT_NIR_); and (6) immersion in water for 168 h after sealing the device edges with a UV‐curable adhesive to prevent water ingress and electrolyte leakage (Figure S12), after which the device retained 100% and 91% of its initial ΔT_vis_ and ΔT_NIR_ values, respectively. Therefore, these results demonstrate that the TriV‐based EC device possesses good cycling durability and environmental tolerance under the tested laboratory aging conditions. In addition, they also prove the stability of the TriV‐based EC electrolyte developed, as no visible electrolyte leakage, bubble formation, or interfacial delamination was observed following any of the aging tests conducted (Figure S13).

Overall, the TriV‐based EC device provides a balanced combination of high visible light transmittance in the bleached state, near‐black coloration and visible–NIR broad‐spectrum modulation upon reduction, low‐voltage operation, fast response, and high cycling and environmental stability, which fairly compares with some of the most recent broadband electrochromic smart windows reported in the literature (Table S4).

### 2.3 Structural Design and Optical Regulation of the ETC Smart Window

**FIGURE 5:**
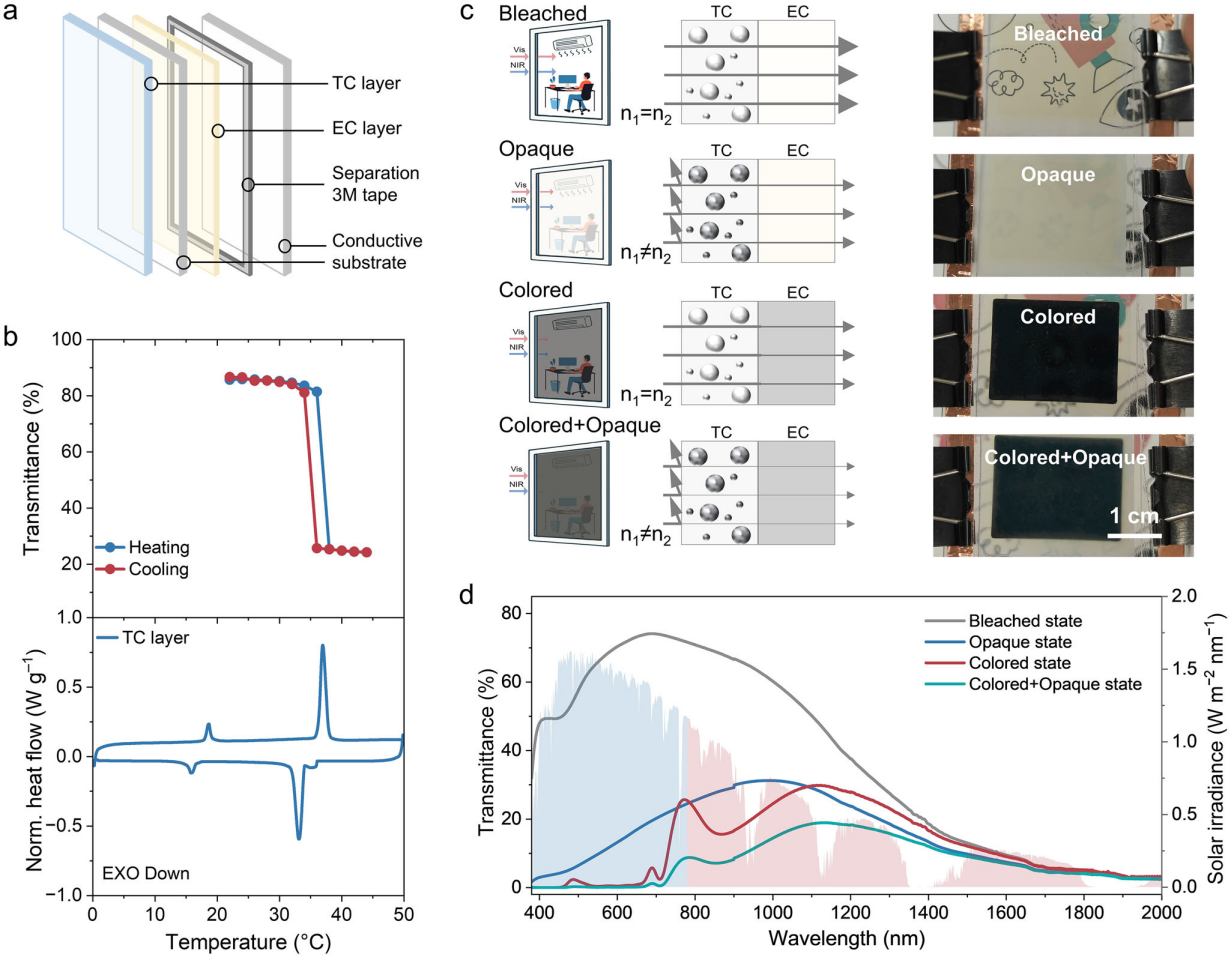
Hybrid electro‐thermochromic device enabling multi‐stimuli‐responsive smart window functionality. (a) Schematic illustration of the multi‐stimuli‐responsive ETC device. (b) (top) Temperature‐dependent variation of transmittance at 550 nm of a paraffin‐based TC film deposited on glass. (bottom) DSC thermogram of a paraffin‐based TC film (exo down, 2°C min^−1^). (c) Different operation modes of the ETC device, for which representative photographs are shown. (d) UV–vis–NIR transmittance spectra of an ETC device in the bleached transparent state (0 V, room temperature), the thermally‐induced opaque state (0 V, ∼55°C), the electroinduced colored state (−1.0 V, room temperature), and the combined colored+opaque state obtained by simultaneous heating and voltage application (−1.0 V, ∼55°C).

For the preparation of the ETC smart window, the multiviologen TriV‐based EC device described above was integrated with a paraffin‐based TC layer (Figure 5a). This layer consisted of paraffin microparticles embedded in a PVA film and fabricated by a blade‐coating process, following a method previously optimized by us (paraffin@PVA) [30]. To this end, a mixture of paraffins (eicosane and docosane, 9:1 w/w) was first dispersed in an aqueous solution of PVA (8 wt.%) and glycerol (2 wt.%), forming a stable emulsion. According to dynamic light scattering (DLS) analysis, this emulsion was primarily composed of microparticles with diameters between 1 and 5 µm, together with a minor submicron population with sizes below 1 µm (Figure S14). The preparation of the TC material was then achieved by doctor blade deposition of three layers onto the non‐conductive side of an ITO glass substrate. Specifically, the two external layers (1st and 3rd deposited layer) were obtained by depositing a PVA/glycerol aqueous solution, while the central layer (2nd deposited layer) was prepared from the paraffin microparticle emulsion, overall forming a thermochromic film. The 3‐layered structure could be observed by scanning electron microscopy (SEM) analysis of the film cross‐section, showing a total film thickness of 77 µm (Figure S15).

The thermoresponsive behavior of our TC film arises from the phase change of the embedded paraffin microparticles, which undergo a sharp solid‐liquid transition at ca. 36 C–37 C [30, 55]. Below the transition temperature, the refractive indices of solid paraffin particles and PVA are closely matched ($n_{\mathrm{solid}}^{\mathrm{paraffin}}$ ∼ $n_{\mathrm{solid}}^{\mathrm{PVA}}$ = 1.51), minimizing light scattering and yielding a transparent state. Upon heating, paraffin melts, reducing its refractive index and creating a significant mismatch with PVA ($n_{\mathrm{liquid}}^{\mathrm{paraffin}}$ ∼ 1.44), giving rise to strong light scattering and resulting in a drastic drop in transmittance. Differential scanning calorimetry (DSC) analysis between 0°C and 50°C of the thermochromic composite confirmed the reversible solid‐to‐liquid and liquid‐to‐solid transitions of the paraffin particles at 37°C and 34°C respectively, matching the temperatures at which the transmittance change is observed (Figure 5b and Figure S16). In addition, weaker endothermic and exothermic processes were observed by DSC at 18.6°C and 15.8°C, respectively, which can be associated to a secondary solid‐solid transition corresponding to the rotator‐crystalline phase change of the paraffin particles. As described previously by us, the additional phase change has a minimal influence on the optical properties of the film, which remains transparent upon further cooling [30].

In agreement with our previous results [30], the paraffin‐based TC layer prepared showed high visible transparency in the initial state at room temperature (AVT ∼ 80% at 22°C) and ample transmittance modulation over the whole solar spectrum upon heating (ΔT_solar_ ∼ 58%, 390–2500 nm). These figures lie close to the best figures reported for hydrogel‐ [19, 20, 21, 22] and ionogel‐based [23, 24, 25] TC smart windows (AVT ∼80%–95%, ΔT_solar_ ∼55%–90%) and surpass the performance of other well‐established TC technologies such as those based on vanadium oxide [16, 17, 18] (AVT < 75%, ΔT_solar_ ∼10‐45%) (Table S5). In addition, our TC system demonstrated fast switching responses (< 70 s) that are comparable with those of the best thermochromic materials (Figure S17 and Table S5), despite the small hysteresis effects observed by DSC when cooling to induce the opaque‐to‐transparent transition (thermal hysteresis width ∼ 3.5°C, Figure 5b). In addition, paraffin@PVA thermochromic layers are highly resistant to operational and environmental conditions, as we have previously demonstrated that their thermochromic reponse is marginally affected by repetitive thermal switching between the transparent and opaque states, prolonged sunlight irradiation under outdoor conditions, combined UV and high‐temperature aging tests, and exposure to low temperatures [30]. Although the main disadvantage of these TC materials is the water sensitivity of its hydrophilic PVA‐based matrix, this issue can be readily overcome by coating with a water‐resistant film, as previously proven by us [30]. In this work, a transparent poly(dimethyl siloxane) (PDMS) protective coating was introduced as a novel proof‐of‐concept moisture‐barrier layer for this type of TC layers, which did not significantly affect their optical transparency. When analyzing the humidity stability of unprotected and protected paraffin@PVA films, clear differences were observed (Figure S18). After aging at 85% relative humidity (RH) and 25°C for 7 days, the unprotected TC layer showed a 36% increase in mass due to water absorption and obvious spectral changes, which resulted in a clear attenuation of its temperature‐responsive optical switching behavior (about 40%). In contrast, the PDMS‐protected TC layer exhibited negligible mass (< 0.5%) and spectral variation after the same humidity‐aging treatment, preserving the thermochromic performance of the paraffin‐based composite.

After integration of the paraffin‐based TC film with a redox‐matched TriV‐based EC device in a single ETC system, four distinct optical modes are enabled, providing responsive and tunable modulation of both visible and NIR light (Figure 5c,d). In the bleached mode achieved at 0 V and room temperature, the device is both colorless and transparent, yielding an AVT of 65.8%; i.e., the same as that of the pure EC film shown above, indicating that the TC layer is not affecting the overall transmittance of the system in its non‐activated state. The transmittance over the whole solar spectrum results in a slightly lower value of T_solar_ = 55.6%, mainly due to the moderate transparency of ITO in the NIR region (T_NIR_ = 44.7%, over 780–2500 nm). The opaque mode is accomplished when the TC coating is thermally activated by heating above paraffin's melting point (ca. 36°C–37°C) while the EC layer remains transparent, passively providing solar shading and glare reduction (AVT = 10.5%, T_solar_ = 16.6%). In the colored mode generated by applying −1.0 V, the redox‐matched TriV‐based gel undergoes electrochemical reduction, achieving a near‐black state while the TC layer remains transparent (AVT = 0.7% and T_solar_ = 10.3%). Finally, in the colored+opaque mode obtained by simultaneously applying −1.0 V and heating the TC layer above its transition temperature, both layers are activated, yielding the lowest transmittance among all operation conditions (AVT = 0.1%, T_solar_ = 5.4%) and reaching light modulation amplitudes of ΔT_solar_ = 50.2% and ΔT_NIR_ = 33.7% for the solar and NIR (780–2500 nm) spectra.

To preliminarily evaluate the scale‐up feasibility and optical uniformity of our hybrid ETC systems, larger 10 × 10 cm^2^ TriV‐based EC devices and paraffin@PVA TC layers were fabricated under laboratory conditions. Both enlarged samples showed clear optical switching, while position‐dependent transmittance spectra exhibited good spatial consistency (Figure S19), suggesting acceptable uniformity of the solution‐processed TC coating and integrated EC gel device. In addition, a preliminary raw‐material cost estimation was performed for a 1 × 1 m^2^ ETC device with a 0.2 mm‐thick EC gel layer and the present three‐layer TC coating structure. The estimated raw‐material cost was 570.4 € m^−2^, mainly corresponding to the components of the TriV‐based EC device (548.7 € m^−2^, Table S6). As we have already reported previously [30], the raw‐material cost of the paraffin@PVA TC layer is very reduced (21.7 € m^−2^, Table S6) when compared to other TC technologies involving more expensive active materials (e.g., thermally‐responsive polymers for hydrogels, liquid crystals) or EC devices requiring costly electrochromes, electrolytes and conductive substrates. Consequently, the incorporation of the TC layer should entail virtually no additional significant cost to EC device fabrication, while delivering substantial functional benefits and enhancing the overall performance and energy‐saving potential of the resulting ETC smart‐window system, as described in detail below.

### 2.4 Thermal Management and Energy‐Saving Performance of the ETC Smart Window

**FIGURE 6:**
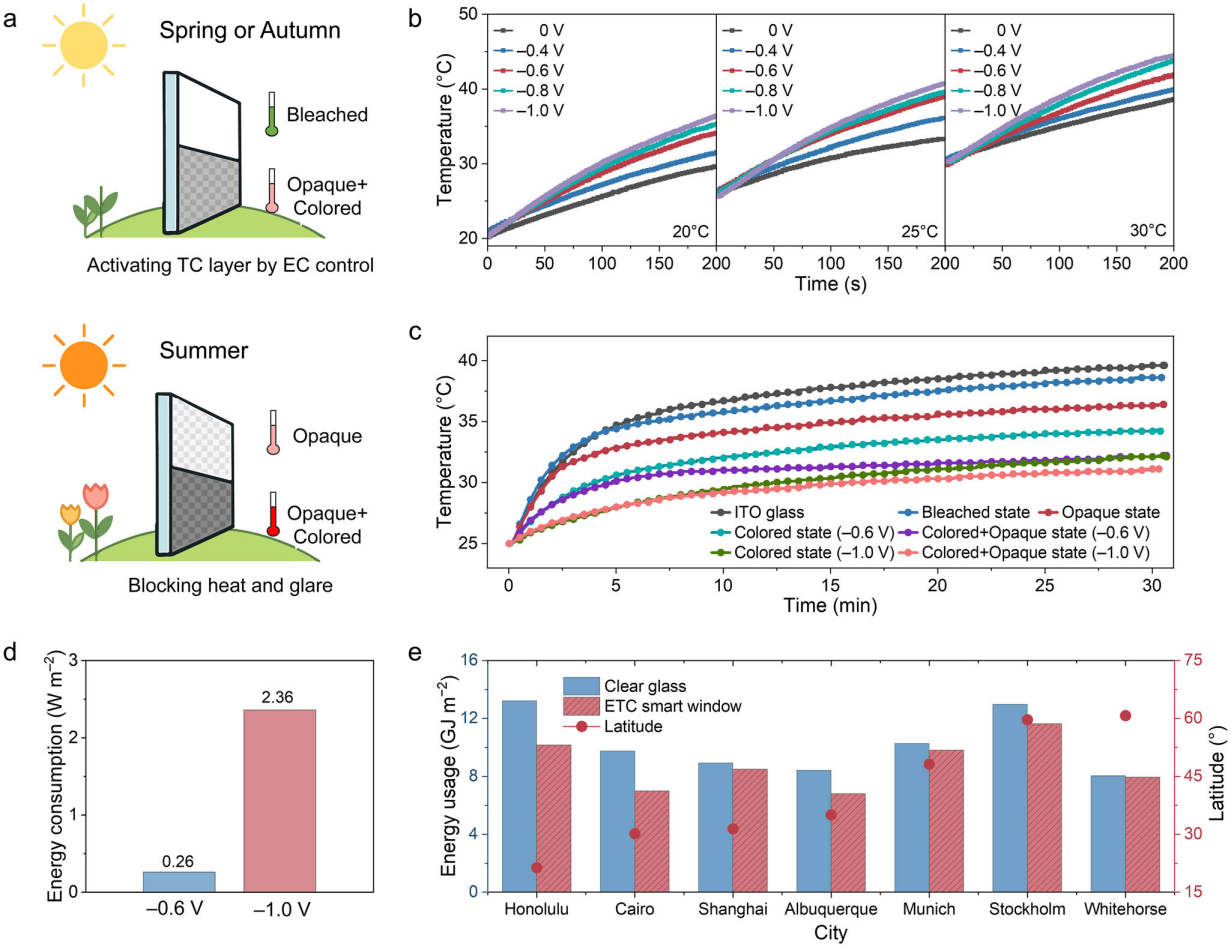
Seasonal adaptability and thermal energy‐saving performance of the multistimuli‐responsive ETC smart window. (a) Schematic illustrations of ETC smart window operation modes in representative seasonal climates. (b) Effect of ambient temperature and applied voltage on the temperature variations achieved at the surface of an ETC smart window after 200 s of irradiation with a solar simulator (AM 1.5G, 1000 W m^−2^). (c) Real‐time temperature rise profiles in an insulated box under different switching modes of the ETC smart window, measured at an ambient temperature of 25°C. (d) Electrical energy consumption of the ETC smart window as a function of applied voltage. (e) Simulated energy consumption of a full‐scale building model in cities of variate climates using clear glass and an ETC smart window.

Apart from offering user‐controlled modulation of solar irradiation, the electroinduced nearly‐black EC layer of the ETC smart window converts the absorbed light into heat, raising the local temperature of the whole device. This tunable photothermal response provides an active means to assist the TC transition without relying on permanently absorbing photothermal additives that compromise visible and NIR transparency when maximum sunlight transmission is desired (e.g., low sunlight intensity and/or temperature). As a result, the EC layer can be used to selectively trigger the melting of paraffin at different ambient temperatures simply by varying the applied voltage, enabling controlled activation of the TC layer with minimal additional energy input (Figure 6a).

To evaluate this effect, the temperature of the ETC device under simulated sunlight (AM 1.5G, 1000 W m^−2^) was monitored for 200 s using a thermocouple. The experiment was performed at applied voltages of 0, −0.4, −0.6, −0.8, and −1.0 V, and at ambient temperatures of 20°C, 25°C, and 30°C. As expected, increasing the applied potential led to higher device temperatures due to enhanced light absorption and photothermal conversion, indicating that the thermochromic response of the hybrid device could be controlled by the user. After 200 s at an ambient temperature of 20°C, the activation of the TC layer was not triggered at any of the voltages applied. Instead, at 25°C, a voltage equal or higher than −0.6 V would be sufficient for melting the paraffin particles. Finally, at 30°C activation occurred even in the absence of an applied voltage, likely due to the non‐negligible intrinsic sunlight absorption of the system across the UV–vis–NIR range (e.g., glass, PC and PVA absorption in the NIR range). Thus, the operational mode of the device can transition from a fully active EC mode (low ambient temperature, −1.0 V) to a completely passive TC mode (high ambient temperature and illumination intensity, 0 V), with intermediate hybrid modes in between (Figure 6b).

To demonstrate that the photothermally‐assisted activation of the TC layer is enhanced by the black electrochromic module selected for our hybrid ETC system, additional experiments were conducted. In the absence of solar irradiation, negligible thermal heating (1°C–2°C) was measured due to the Joule effect generated when passing an electric current through the EC device to trigger its electrochromic response (Figure S20). Therefore, this result proves that the photothermal heating caused by sunlight absorption of the colored state of the electrochromic module is required to promote the photothermal activation of the thermochromic layer. In addition, we evaluated the photothermal effects in single‐color electrochromic devices containing each of the separate MV, PV and M‐bisV species. At the maximum voltage applied (−1.0 V), a two‐fold decrement in photothermal heating associated with viologen coloration was measured for single‐color electrochromic devices (3°C–4°C) compared to the multiviologen black electrochromic system (8.1°C) (Figure S21). Furthermore, none of the single‐color electrochromic devices produced sufficient photothermal heat at an applied voltage of −0.6 V to promote the activation of the thermochromic layer for an external temperature of 25°C, in contrast to the behavior observed for the TriV‐based black electrochromic system. Overall, this data unambiguously demonstrates that the selection of a black electrochromic layer strongly maximizes photothermal effects, favoring the synergistic operation of hybrid ETC smart windows.

To quantitatively evaluate the thermal regulation capabilities of ETC devices, controlled indoor solar‐light induced heating tests were conducted using a custom‐built insulated test box and a solar simulator (AM 1.5 G, 1000 W m^−2^), reproducing the intensity and spectral features of sunlight (Figure S22). This setup was designed to provide a reproducible comparison of the relative heat‐gain regulation capability of different window states under identical irradiation and testing conditions. By keeping the chamber geometry, irradiation intensity, and temperature‐measurement configuration consistent, the thermal‐box experiment allows the effects of the bleached, colored, opaque, and colored+opaque states to be directly compared. The chamber consisted of a 10 × 10 × 10  cm^3^ expanded polystyrene cube, with 2 cm‐thick walls, and a 3 × 3  cm^2^ window aperture. A double‐glazed window assembly was mounted at the opening, comprising the device as the outer pane and a 5 mm glass substrate as the inner pane, separated by a 2 cm air gap. A thermocouple placed at the center of the chamber recorded the internal temperature in real time during continuous irradiation with a solar simulator. Notably, an ITO glass was included as a reference in the thermal‐regulation tests, as its strong NIR reflectance provides a meaningful benchmark for evaluating heat‐blocking performance. For conditions requiring selective evaluation of the EC response (bleached or colored state), an independent EC device was used instead of the ETC window to avoid unintended activation of the TC layer under solar illumination.

Figure 6c and Figure S23 show the temperature increase measured inside the insulating box for each window and operation mode during solar irradiation for 30 min, after which a steady state situation was essentially obtained in all cases. Using an ITO glass, the solar heating gain produced a temperature increase (ΔTemperature) of 14.6°C after 30 min. Notably, when an ETC window was used instead, a comparable ΔTemperature of 13.7°C was observed in its bleached state, owing to its high sunlight overall transmittance despite the non‐negligible light absorption and reflection by the device layers at 0 V; i.e., allowing for efficient passive heating indoors in cold seasons. In contrast, significant solar heating reduction was measured for the different active operation modes of the ETC device. In the opaque mode, its TC layer effectively scattered both visible and NIR light, lowering ΔTemperature to 11.4°C (∼17% decrement compared to the bleached state). The colored mode, achieved by applying –0.6 V to the EC layer, provided stronger thermal blocking with a ΔTemperature of 9.2°C (∼33% decrement), an effect that was further enhanced by increasing the voltage to −1.0 V (ΔTemperature = 7.2°C, ∼47% decrement). The most significant reduction was observed in the colored+opaque configuration, where EC absorption and TC scattering worked in tandem. This resulted in ΔTemperature values of 7.2°C at −0.6 V and 6.1°C at −1.0 V, reaching ∼47%–55% decrement in solar heat gain.

Interestingly, an equivalent modulation in solar heating (ΔTemperature = 7.2°C) was achieved by operating only the EC layer of the hybrid device at high voltage (colored state at −1.0 V) or through its synergistic electro‐thermochromic response at lower voltage (colored+opaque state at −0.6 V). This result highlights the potential of ETC windows to block solar radiation efficiently while minimizing power consumption. Based on the current density measured during 30 min of operation of the ETC device at −0.6 and −1.0 V, we estimated the average electrical energy consumption to be 0.26 and 2.36 W m^−2^ at these two conditions, respectively, indicating that operating costs could be reduced by ∼90% compared to pure EC windows without compromising energy efficiency performance (Figure 6d and Figure S24). For a model smart window area of 1 × 1 m^2^ operated in Barcelona during July (∼10 h of sunlight per day, average temperature above 25°C), this would translate to an approximate reduction of 0.65 kWh in electrical energy consumption, underscoring the added benefit of photothermal‐assisted, combined EC and TC operation in hybrid ETC devices.

This synergistic effect represents only one of the key advantages of the present ETC device over previously reported hybrid electro‐thermochromic smart windows [37, 38, 39, 40, 41, 42, 43] (Table S7). While exhibiting comparable initial visible light transmittance, solar transmittance modulation and thermal‐regulation capability to those precedents (AVT ∼ 70%–80%, ΔT_solar_ ∼ 50%–70%, and ΔTemperature ∼ 5.0°C–14.0°C), our ETC device provides several additional advantages: (1) it operates at the lowest voltage (−1.0 V) and, even better, it remains in the bleached state at 0 V, in contrast to most previously reported systems that require an external bias to reach the bleached state of the EC component (1.0–30 V); (2) it employs a paraffin@PVA thermochromic layer, which features a simpler architecture, lower cost, and greater environmental stability than the hydrogel‐based TC components used in most prior systems; and (3) by tuning the photothermal effects generated by the EC unit upon voltage variation, the temperature at which the transparent‐to‐opaque activation of its TC layer occurs can be precisely and dynamically adjusted to the specific demands of different geographical regions and times of the year. Combined with the power consumption reduction arising from the photothermally‐assisted activation of the TC layer upon sunlight absorption by the EC device, these attributes position our ETC system as a highly attractive smart‐window technology for the cost‐effective management of solar heat gain, offering a unique combination of low‐power operation, low material cost, and efficient solar heat modulation.

To further investigate the energy efficiency gain provided by our hybrid ETC devices in terms of solar heating modulation, building energy simulations were carried out in EnergyPlus using a standard office building model equipped with ETC or clear glass windows (Figure S25). In all simulations, the ETC window was assumed to remain in the bleached state below 25°C and to switch into the colored+opaque state above 25°C under an applied voltage of −1.0 V. The simulated annual heating and cooling loads computed for both cases and across different climatic regions are given in Figure 6e and Figure S26 and Table S8. In all cases, the ETC window consistently reduces the total energy load compared to clear glass, confirming its capability for adaptive solar regulation. In tropical and subtropical climates such as in Honolulu and Cairo, where cooling demand dominates, the ETC window achieves remarkable energy savings of 3.05 GJ m^−2^ and 2.75 GJ m^−2^, respectively, by effectively suppressing solar heat gain. In temperate regions (e.g., Shanghai and Albuquerque), the energy reduction remains substantial, reaching 0.43–1.63 GJ m^−2^, owing to balanced cooling and heating loads throughout the year. For high‐latitude cold climates such as Munich, Stockholm, and Whitehorse where heating demand becomes dominant, yet the ETC window still delivers measurable benefits with energy reductions ranging from 0.09 to 1.34 GJ m^−2^. The overall improvement in energy efficiency demonstrates that the ETC window can effectively balance cooling energy savings and thermal comfort, representing a practical and scalable approach for sustainable building design.

## 3 Conclusions

In this work, we developed electro‐thermochromic smart windows that deliver ample, multimode modulation of solar irradiation at low operation costs, addressing several fundamental limitations of traditional electro‐ and thermochromic systems. The hybrid ETC devices comprise two distinct active components: (1) an electrochromic layer composed of three complementary‐colored, redox‐ and switching time‐matching viologen molecules, which enables on‐demand black coloration with extremely low visible light transmittance (∼ 0.7%), rapid and homogeneous switching (∼ 10–20 s) and excellent cycling stability; and (2) a low‐cost paraffin‐based thermochromic film that becomes opaque upon heating, efficiently blocking visible and near‐infrared radiation. When combined in a single device, these components offer several synergistic advantages. First, they feature voltage‐tunable, multimode operation, allowing both active and passive regulation of solar transmission and enabling users to balance energy savings with additional needs such as glare and privacy control. Second, the strong solar absorption of the electrochromic colored state promotes photothermal activation of the thermochromic layer, increasing optical modulation while reducing electrical energy consumption by approximately an order of magnitude. This is accomplished through modulable photothermal conversion, without requiring permanently absorbing photothermal layers, which detrimentally filter incident light during cold or low sunlight intensity conditions. Third, the complementary spectral response achieved through simultaneous electro‐ and thermochromic activation yields nearly complete suppression of solar transmittance across the full spectrum (∼5.4%). As a result of these combined effects, ETC windows achieved an 8.5°C reduction in solar heat gain under simulated sunlight on hot days, demonstrating substantial potential for thermal management and building energy efficiency. Energy‐use simulations further indicate up to a 23% reduction in annual building energy consumption in low latitude regions (e.g., Honolulu), confirming the practical energy‐saving potential of this hybrid platform. Overall, this work establishes a new paradigm for smart window technologies by integrating voltage‐controlled, black electrochromism with photothermal‐assisted thermochromism. This design strategy enabled us not only to achieve superior performance but also to establish a set of design principles for the rational development of optimized ETC smart windows, in which the complementary operation of both electro‐ and thermochromic technologies is engineered to optimize solar heating management and energy savings in buildings.

## 4 Experimental Section

### 4.1 Materials

All reagents and solvents were used as received without further purification. 4,4′‐Bipyridine, 1,3‐diiodopropane, iodomethane, ammonium hexafluorophosphate, ferrocene (Fc), lithium bis(trifluoromethylsulfonyl)imide (LiTFSI), tetrabutylammonium hexafluorophosphate (TBAPF_6_), acetonitrile, diethyl ether, and acetone were purchased from Sigma–Aldrich. 1‐Ethyl‐3‐methylimidazolium bis(trifluoromethylsulfonyl)imide ([EMIM][TFSI]) was obtained from Solvionic. Propylene carbonate (PC) was supplied by Carlo Erba Reagents. Eicosane (> 98%) and poly(methyl methacrylate) (PMMA, M_w_ = 350,000) were obtained from TCI. Docosane (99%) and glycerol (> 99.5%) were purchased from Merck. Poly(vinyl alcohol) (PVA, 40–88) was acquired from Kuraray. Polydimethylsiloxane (PDMS; SYLGARD 184 Silicone Elastomer Kit) was purchased from Dow Chemical Company. Methyl viologen dichloride was purchased from Sigma–Aldrich and converted to the bis(hexafluorophosphate) salt (MV) via ion exchange with NH_4_PF_6_. The methyl‐bridged bisviologen (M‐bisV), phenyl viologen (PV), and monoheptyl viologen (Mono‐HV) were synthesized following previously reported procedures [49, 50, 54]. Aniline, methanol, 1‐bromoheptane, and 2,4‐dinitrochlorobenzene were purchased from Shanghai Macklin Biochemical Co., Ltd. Indium tin oxide (ITO) glass substrates (thickness: 1.1 mm; sheet resistance: ∼10 Ω sq^−1^) were purchased from Techinstro. Indium tin oxide‐coated polyethylene terephthalate (ITO‐PET) substrate (thickness: 0.175 mm; sheet resistance: 45 Ω sq^−1^) was purchased from Zhuhai Kaivo Optoelectronic Co., Ltd.

### 4.2 Preparation of Viologen‐Based EC Gels

Viologen‐based EC gels were prepared by dissolving the viologen species together with Fc (20 mmol L^−1^) and [EMIM][TFSI] (0.2 mol L^−1^) in a PMMA/PC solution, where the mass ratio of PMMA to PC was maintained at 1:10. For the single‐viologen EC gel, MV or PV was used at 20 mmol L^−1^, while M‐bisV was used at 10 mmol L^−1^, equivalent to 20 mmol L^−1^ bipyridinium units. For TriV‐based EC gel, 20 mmol L^−1^ MV, PV, and 10 mmol L^−1^ M‐bisV were added at EC gel, corresponding to a molar ratio of MV: PV: M‐bisV = 2: 2: 1. The mixture was stirred for 2 h at room temperature and subsequently heated at 70°C for 2 h to ensure complete dissolution and homogeneous mixing.

### 4.3 Preparation of PVA/Glycerol Solution

While stirring, 20 g of PVA 40–88 were added to a round‐bottom flask containing 180 g of distilled water at room temperature. The mixture was stirred at 85°C until the complete dissolution of the polymer. Next, 50 g of a glycerol solution (10 wt.% in water) were added and mixed homogeneously by stirring.

### 4.4 Preparation of Paraffin Emulsion

720 mg of eicosane and 80 mg of docosane (9:1 w/w) were added to a 50 mL beaker equipped with a cross‐shaped magnetic stirrer and heated to 60°C. In parallel, 32 g of PVA/glycerol solution were poured into another 50 mL beaker and heated to 60°C under stirring. Once reached the temperature, the PVA/glycerol solution was poured into the beaker containing the melted paraffins while stirring. Then, the mixture was stirred at 700 ppm for 10 min, forming a pre‐emulsion. Afterward, this mixture was emulsified using an ultrasonic sonifier operating at 70% amplitude (Branson 450 D Sonifier, 400 W‐20 kHz, equipped with disruptor horn and 13 mm flat tip) during 90 s, in cycles of 15 s of sonication and mixing manually between cycles to ensure homogeneous emulsification. Finally, the emulsion was poured in a centrifuge tube and stored for a minimum of 1 day before using, to get rid of air bubbles.

### 4.5 Fabrication of Paraffin@PVA Thermochromic Film

The paraffin@PVA thermochromic film was fabricated following a previously reported procedure with slight modifications [30]. Using a Zehntner ZAA 2300 doctor blade equipment, the following layers were deposited on an ITO glass substrate (40°C, speed 3 mm s^−1^, blade height 400 µm): (1) PVA/glycerol solution, (2) paraffin emulsion, (3) PVA/glycerol solution. Each layer was dried for 30 min at 40°C before casting the next one. After formation of the paraffin@PVA thermochromic film, the PDMS prepolymer and curing agent were mixed at a mass ratio of 10:1 and blade‐coated onto the surface of the thermochromic layer. The resulting PDMS‐coated film was then cured at 50°C for 12 h.

### 4.6 Fabrication of Viologen‐Based EC Devices and ETC Devices

The viologen‐based EC devices were assembled by sandwiching the EC gels between two pieces of ITO glass, with thickness set at 0.2 mm using double‐sided tape. Unless otherwise specified, the effective area of the EC device was maintained at 3.14 cm^2^ by punching the double‐sided tape with a circular mold (diameter = 2.0 cm). For the fabrication of ETC devices, the same procedure was followed, except that one of the ITO glasses was pre‐coated with the paraffin@PVA film in the non‐ITO‐coated side.

### 4.7 Characterization

Dynamic light scattering measurements of paraffin emulsions were obtained using a Malvern Zetasizer zs. SEM images of the paraffin‐PVA film cross section was registered with a FEI Quanta 650 ESEM microscope applying a 5 kV voltage. The film samples were metalized with a layer of about 5 nm of Pt prior to SEM imaging. Differential scanning calorimetry measurements (DSC) of these films were obtained using a TA instruments Q20. A temperature ramp rate of 2°C min^−1^ was used for all samples. The viscosity of the EC gel electrolytes was measured using an Anton Paar rheometer (MCR 102e) to evaluate their flow and mechanical properties. Their ionic conductivity was obtained through AC impedance testing (EIS) using a CHI660E electrochemical workstation and calculated using the following Equation (1):

(1) $$\sigma =L\;/\;(R_{b}A)\;$$

where *L*, *R_b_*_,_ and *A* represent the EC gel thickness, bulk resistance, and effective area, respectively.

Cyclic voltammetry experiments were performed using a VSP100 potentiostat (BioLogic) controlled by EC‐Lab V9.51 software, in three‐ (working electrode: glassy carbon; counter electrode: Pt; reference electrode: Ag/AgCl) or two‐electrode (EC and ETC devices with ITO‐glass electrodes) systems. Before measurements, the solutions were degassed with N_2_ for 10 min to ensure an inert atmosphere. From voltammograms recorded at different scan rates (*ν*), the corresponding ion diffusion coefficient (*D*) was calculated by the following Randles‐Sevcik Equation (2):

(2) $$i_{p}=2.69\times 10^{5}n^{3/2}AC\;D^{1/2}\;v^{1/2}$$

where *i_p_* is the peak current, *n* is the number of electrons, *A* is the effective electrode area, and *C* is the concentration of the bulk solution. For spectroelectrochemical measurements, in situ electrochemical potentials were applied using a VSP100 potentiostat (BioLogic) controlled by EC‐Lab V9.51 software, synchronized with a Hamamatsu L10290 spectrophotometer. All measurements were conducted in 1‐mm pathlength quartz cuvettes, employing a platinum mesh working electrode, a Pt wire counter electrode, and an Ag/AgCl reference electrode. Before measurements, the solutions were degassed with N_2_ for 10 min to ensure an inert atmosphere.

Optical transmittance of the EC and ETC devices at different operation conditions were measured using Agilent Cary 60 (380–1000 nm) and Jasco U‐770 (300–2500 nm) spectrophotometers, with the devices driven by an external power supply. Thermochromic performance was evaluated using a custom‐made temperature‐controlled holder (SAPPHIRE 360), consisting of two joinable sapphire windows mounted in copper plates, with the temperature increased by applying an external voltage. Long‐term stability tests over 1075 consecutive switching cycles were conducted using a Hamamatsu L10290 spectrophotometer coupled with a VSP100 potentiostat (BioLogic). From optical transmittance data, several performance parameters of the EC and ETC devices were determined. The transmittance of the devices in the luminous (390–780 nm, AVT), solar (380–2500 nm, T_solar_) and near‐infrared (780–2500 nm, T_NIR_) ranges and their modulation under different modes of operation were calculated as follows using Equations ((3), (4), (5)):

(3) $$\mathrm{AVT}(\%),T_{\mathrm{solar}}(\%)\;\mathrm{and}\;T_{\mathrm{NIR}}(\%)=\frac{\int G(\lambda )T(\lambda )d\lambda }{\int G(\lambda )d\lambda }\;100$$

(4) $$\Delta T_{\mathrm{solar}}{=T}_{\mathrm{solar}}^{\mathrm{Non}-\mathrm{activated}}\;{-T}_{\mathrm{solar}}^{\mathrm{Activated}}$$

(5) $$\Delta T_{\mathrm{NIR}}{=T}_{\mathrm{NIR}}^{\mathrm{Non}-\mathrm{activated}}\;{-T}_{\mathrm{NIR}}^{\mathrm{Activated}}$$

where T(λ) represents the measured wavelength‐dependent transmittance normalized to 1, and G(λ) corresponds to (1) the CIE luminous efficiency function for photopic vision for the calculation of the AVT, and (2) the solar irradiance spectrum ASTM G173‐03 Global Tilted 37° between 380 and 2500 nm for T_solar_ and between 780 and 2500 nm for T_NIR_. The CIE Lab color space coordinates (L^*^, a^*^ and b^*^) of the bleached and colored states of EC devices were determined from optical transmittance data using Cary WinUV Color software, while chroma (*C^∗^*) values were calculated using Equation (6):

(6) $$C^{*}=\sqrt{a^{*2}+b^{*2}}$$

where a* and b* represent the color axes from green to red and blue to yellow, respectively. Finally, the coloration efficiency (CE) of EC devices was calculated using Equation (7):

(7) $$\mathrm{CE}=\Delta \mathrm{OD}/\Delta Q=[\log(T_{b}/T_{c})]/(Q\;\cdot \;A)\;$$

where *A* is the effective electroactive area of the device, *Q* is the charged injected to promote coloration, and *T_b_* and *T_c_* refer to the transmittance in the bleached and colored states, respectively.

The light source used in energy efficiency measurements was a solar simulator (Abet SunLite 11002) equipped with an AM1.5G filter, providing 1000 W m^−2^ irradiance. Indoor temperature regulation in the cabin model was measured using a thermocouple thermometer (TASI TA612C) coated with white paste to reflect light.

The extended electrochemical cycling stability of the TriV‐based EC device (11000 cycles) was evaluated using an electrochromic cycling test system (KV‐EC‐7500, Zhuhai Kaiwei, China). Solar aging tests were conducted under a 300 W solar simulator with an AM1.5G filter, providing 1000 W m^−2^ irradiance (Perfectlight Technology Co., Ltd., China). For the coupled simulated solar‐aging/switching test, the device was exposed to simulated solar irradiation while being periodically switched using the electrochromic cycling test system. UV‐aging tests were performed using a 365 nm LED‐UV light source (UVGO, China; 133 W m^−2^), and the samples were irradiated for 48 h. The UV irradiance at the sample position was measured using a UV radiometer (SK‐8203, SNAKOL, China). High‐temperature aging was carried out in an electric thermostatic blast drying oven (DHG‐9075A, Shanghai Yiheng Instruments Co., Ltd., China) at 60°C for 7 days. Low‐temperature aging was performed in a low‐temperature freezer (DW‐40L206, AUCMA, China) at −40°C for 7 days. Humidity‐aging tests were conducted in a constant temperature and humidity chamber (LHS‐50CL, Shanghai Yiheng Scientific Instrument Co., Ltd./Bluepard Instruments, China) at 85% RH and 25°C for 7 days. Water‐aging tests were performed by immersing the edge‐sealed EC device in water for 7 days.

### 4.8 Simulations of Energy Consumption

Building energy simulations were performed using EnergyPlus software to evaluate the impact of window optical and thermal properties on building energy performance. A single‐story office building (27.69 m × 18.46 m × 3.05 m) with a roof size of 28.89 m × 19.66 m × 3.31 m was modeled, containing ten windows (1.83 m × 1.52 m each; six on the longer sides and four on the shorter sides) and one door (1.83 m × 2.13 m). The south‐ and west‐facing windows were equipped with either ETC smart windows or conventional clear glass for comparison. Typical meteorological year (TMY) data from seven representative global cities were used to capture diverse climatic conditions. The ETC window was modeled as a double‐glazed system with temperature‐triggered optical and thermal modulation, activating when the environment temperature exceeded 25°C.

## Author Contributions

The manuscript was written through contributions of all authors. All authors have given approval to the final version of the manuscript. **Xilu Wu**: Writing – original draft, Methodology, Formal analysis, Investigation, Conceptualisation, Data Curation, Validation, Funding acquisition. **Lorenzo Vallan**: Writing – original draft, Methodology, Formal analysis, Investigation, Conceptualisation, Data Curation. **Hao Li**: Methodology, Formal analysis, Conceptualisation, Data Curation. **Jaume Ramon Otaegui**: Methodology, Investigation, Validation. **Silvia Mena**: Methodology, Investigation, Validation. **Weizhong Jiang**: Writing – review & editing. **Hongzhi Wang**: Writing – review & editing, Funding acquisition. **Jonathan C. Barnes**: Writing – review & editing, Funding acquisition, Resources. **Daniel Ruiz‐Molina**: Writing – review & editing, Funding acquisition. **Kerui Li**: Writing – review & editing, Conceptualisation, Funding acquisition. **Jordi Hernando**: Writing – review & editing, Conceptualisation, Funding acquisition, Resources. **Claudio Roscini**: Writing – review & editing, Supervision, Conceptualisation, Funding acquisition, Resources. **Gonzalo Guirado**: Writing – review & editing, Supervision, Conceptualisation, Funding acquisition, Resources.

## Conflicts of Interest

The authors declare no conflicts of interest.

## Supporting information




**Supporting File 1**: advs77765‐sup‐0001‐SuppMat.pdf.

**Supporting File 2**: advs77765‐sup‐0002‐MovieS1.mp4.

## Data Availability

Relevant data supporting this study are available within the article and the Supporting Information file.
