# Supplementary material for: Synergistic Effects in Multistate Black Electro‐Thermochromic Smart Windows for Energy‐Efficient Modulation of Solar Heating
Source: Adv Sci (Weinh). 2026 Sep 23:e77765. Online ahead of print. doi: 10.1002/advs.77765 (PMC13598692; doi:10.1002/advs.77765)
Supplement: Supplementary file 1 [file ADVS-9999-e77765-s002.pdf]

Supporting Information

Synergistic effects in multistate black electro-thermochromic smart windows for energy-efficient modulation of solar heating

Xilu Wu,[1] Lorenzo Vallan,[2] Hao Li,[1] Jaume Ramon Otaegui,[2,3] Silvia Mena,[3] Weizhong Jiang,[1] Hongzhi Wang,[1] Jonathan C. Barnes,[4] Daniel Ruiz-Molina,[2] Kerui Li,[1] Jordi Hernando,[3*] Claudio Roscini,[2*] Gonzalo Guirado[3*]*

Mr. Xilu Wu, Mr. Hao Li, Prof. Weizhong Jiang, Prof. Hongzhi Wang, Prof. Kerui Li

[1] State Key Laboratory of Advanced Fiber Materials, College of Materials Science and Engineering, Donghua University, Shanghai 201620, China.

E-mail: likr@dhu.edu.cn

Dr. Lorenzo Vallan, Prof. Daniel Ruiz-Molina, Dr. Claudio Roscini

[2] Catalan Institute of Nanoscience and Nanotechnology (ICN2), CSIC and The Barcelona Institute of Science and Technology (BIST), Campus UAB, Bellaterra, Barcelona 08193, Spain.

E-mail: claudio.roscini@icn2.cat

Dr. Jaume Ramon Otaegui, Dr. Silvia Mena, Prof. Jordi Hernando, Prof. Gonzalo Guirado

[3] Departament de Química, Universitat Autònoma de Barcelona, 08193 Cerdanyola del Vallès, Spain.

E-mail: jordi.hernando@uab.cat, gonzalo.guirado@uab.cat

Prof. Jonathan C. Barnes

[4] Department of Chemistry, Washington University in St. Louis, St. Louis, Missouri 63130, United States.

This PDF file includes:

Supporting Figures S1 to S26

Supporting Tables S1 to S8

Supporting Movie S1 caption

References section

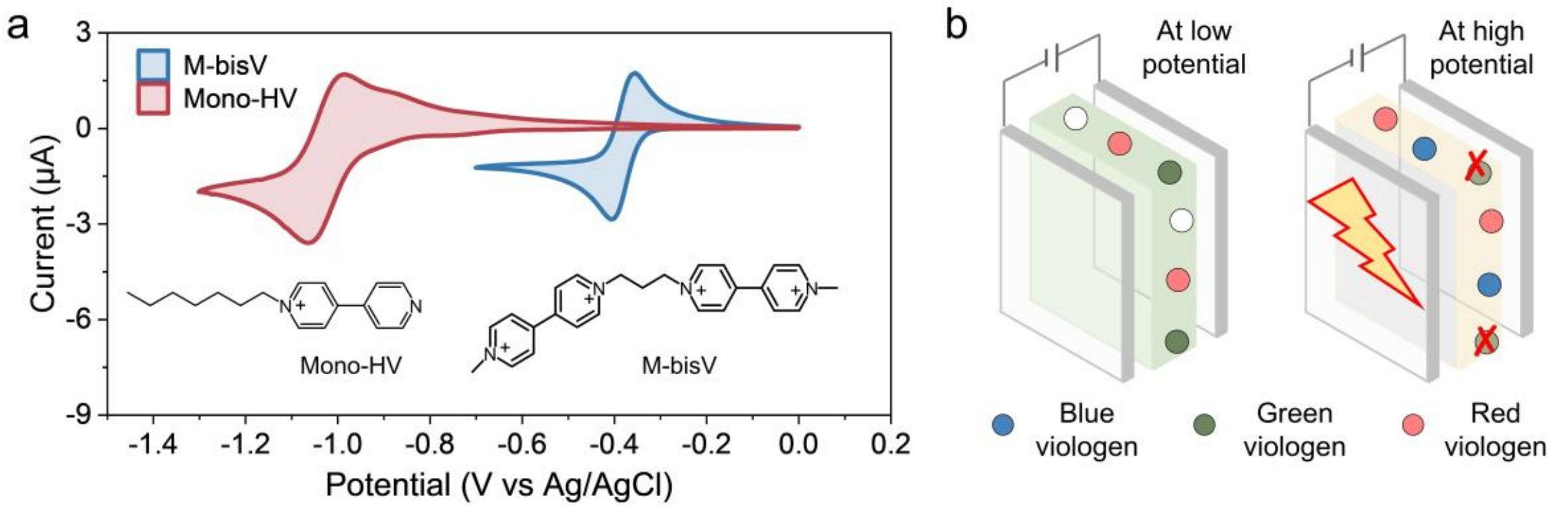

Figure S1. (a) Cyclic voltammograms of solutions of Mono-HV (4 mmol L^{-1}) and M-bisV (2 mmol L^{-1}) in PC/0.2 mol L^{-1} [EMIM][TFSI], measured using a three-electrode cell (working electrode: glassy carbon; counter electrode: Pt; reference electrode: Ag/AgCl; scan rate: 0.01 V s^{-1}). (b) Schematic depiction of color inconsistency and device failure caused by redox mismatch in conventional multiviologen EC devices, which would have occurred if Mono-HV had been used as red viologen electrochrome instead of M-bisV.

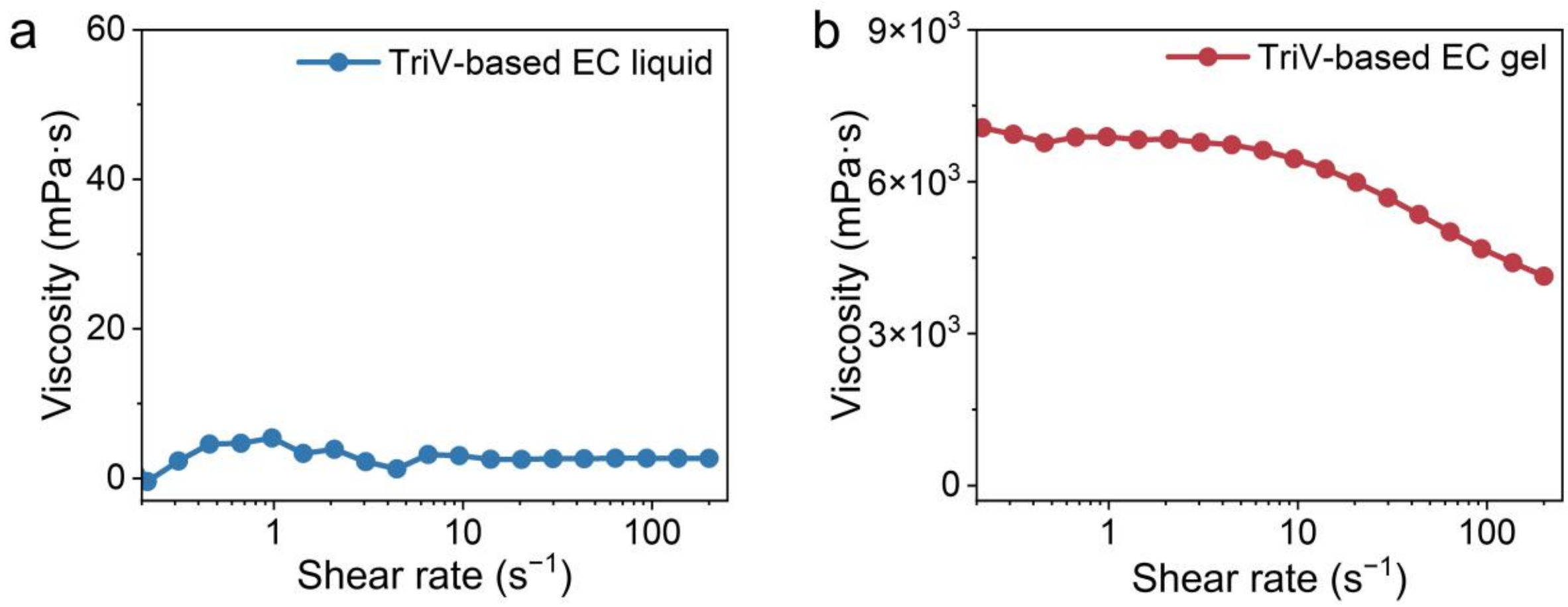

Figure S2. Viscosity profiles of (a) the TriV-based EC liquid (containing 20 mmol L^{-1} MV, PV, Fc, and 10 mmol L^{-1} M-bisV in a solution of [EMIM][TFSI], and PC at a mass ratio of 1:10), and (b) the TriV-based EC gel.

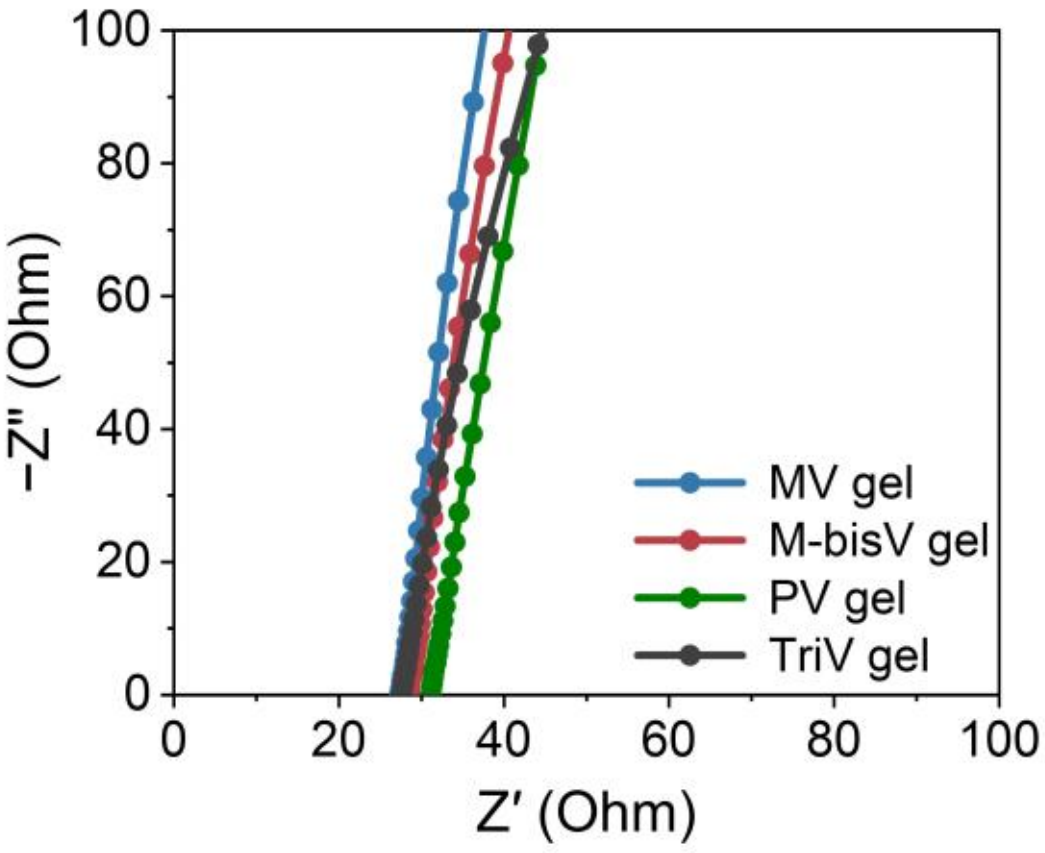

Figure S3. Electrochemical impedance spectra of the viologen-based EC gels (thickness ~ 0.02 cm, area = 1.71 cm^2) measured in an ITO glass/EC gel/ITO glass cell.

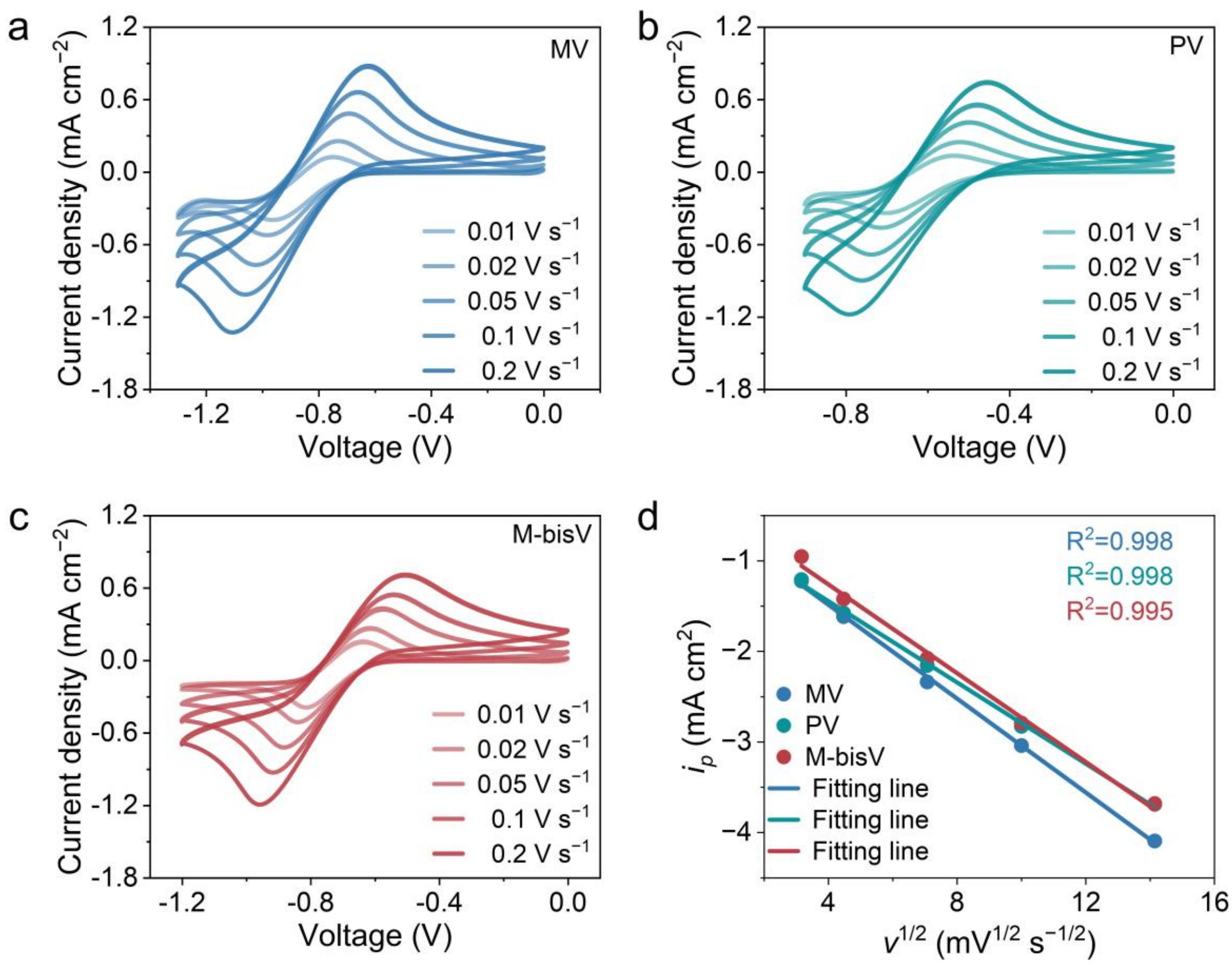

Figure S4. CV curves of (a) MV-, (b) PV-, and (c) M-bisV-based EC gels at scan rates of 0.01, 0.02, 0.05, 0.1, and 0.2 V s^{-1} (working electrode: ITO; counter electrode: ITO). (d) Linear fit of peak current densities (i_p) versus the square root of scan rates ($v^{1/2}$).

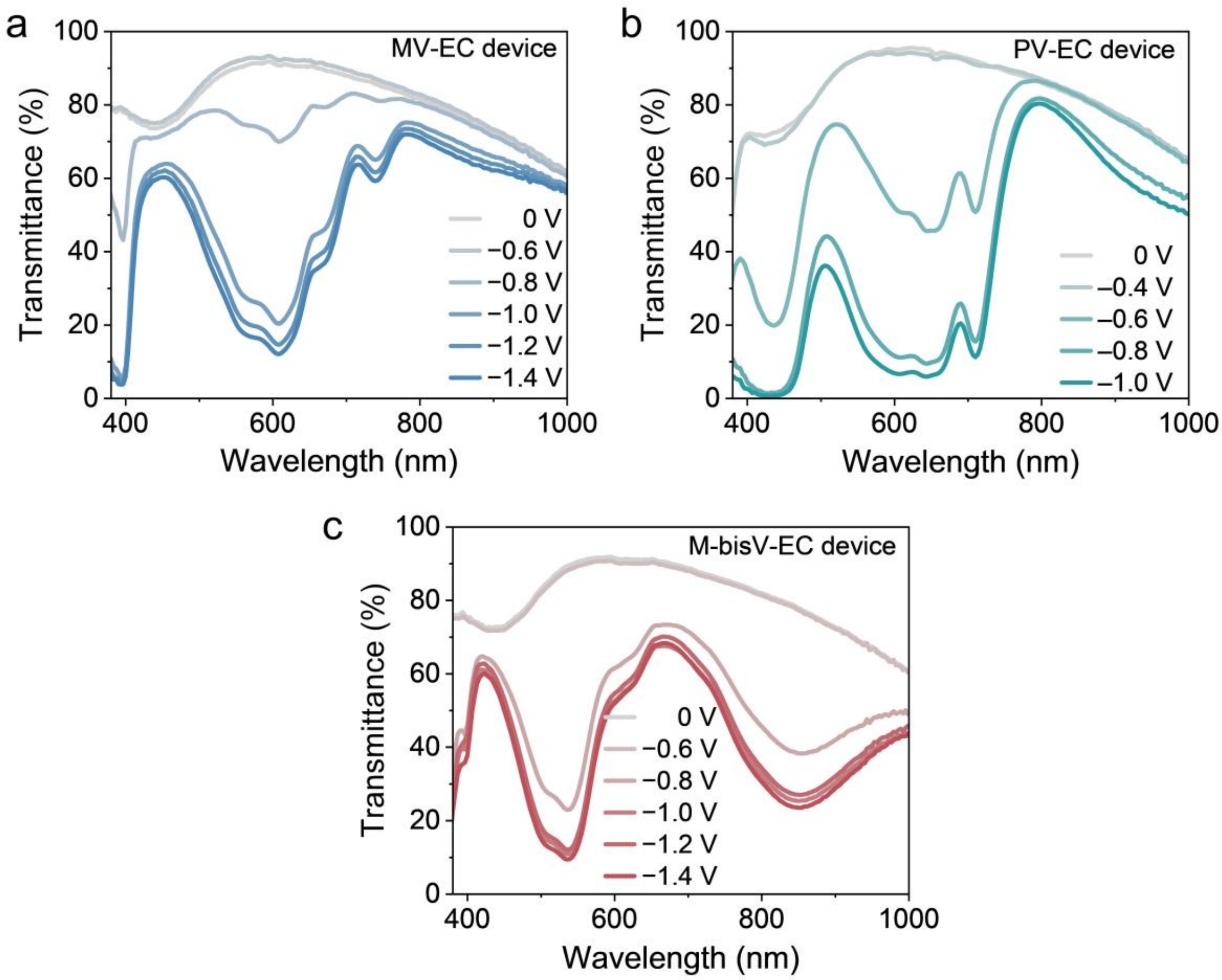

Figure S5. UV-vis transmittance spectra of (a) MV-, (b) PV-, and (c) M-bisV-based EC devices under different voltages.

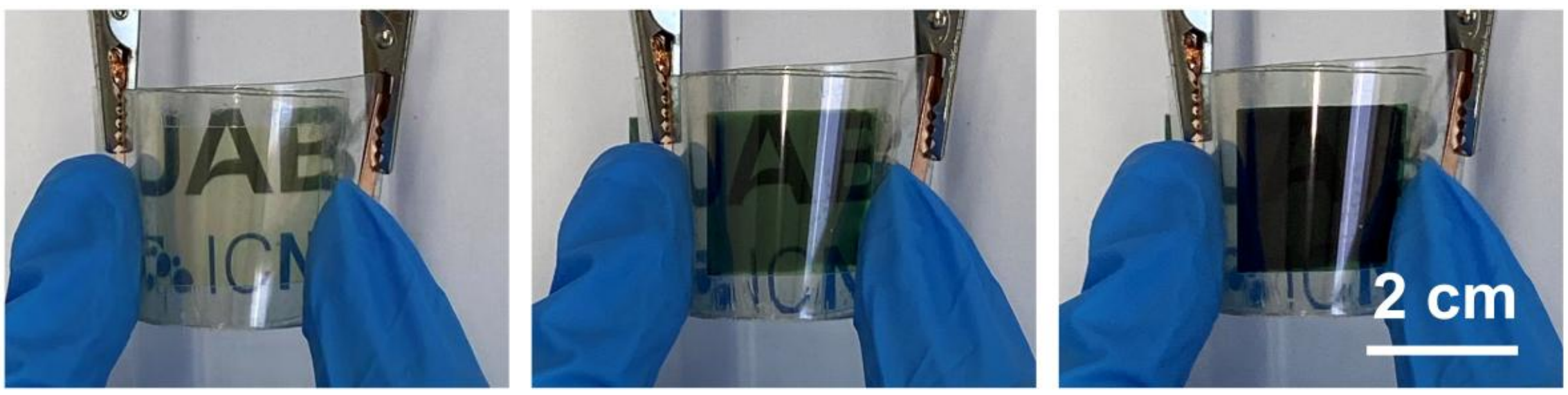

Figure S6. Photographs of a flexible EC device under different applied voltages, which was fabricated by confining the TriV-based gel within ITO-PET electrodes.

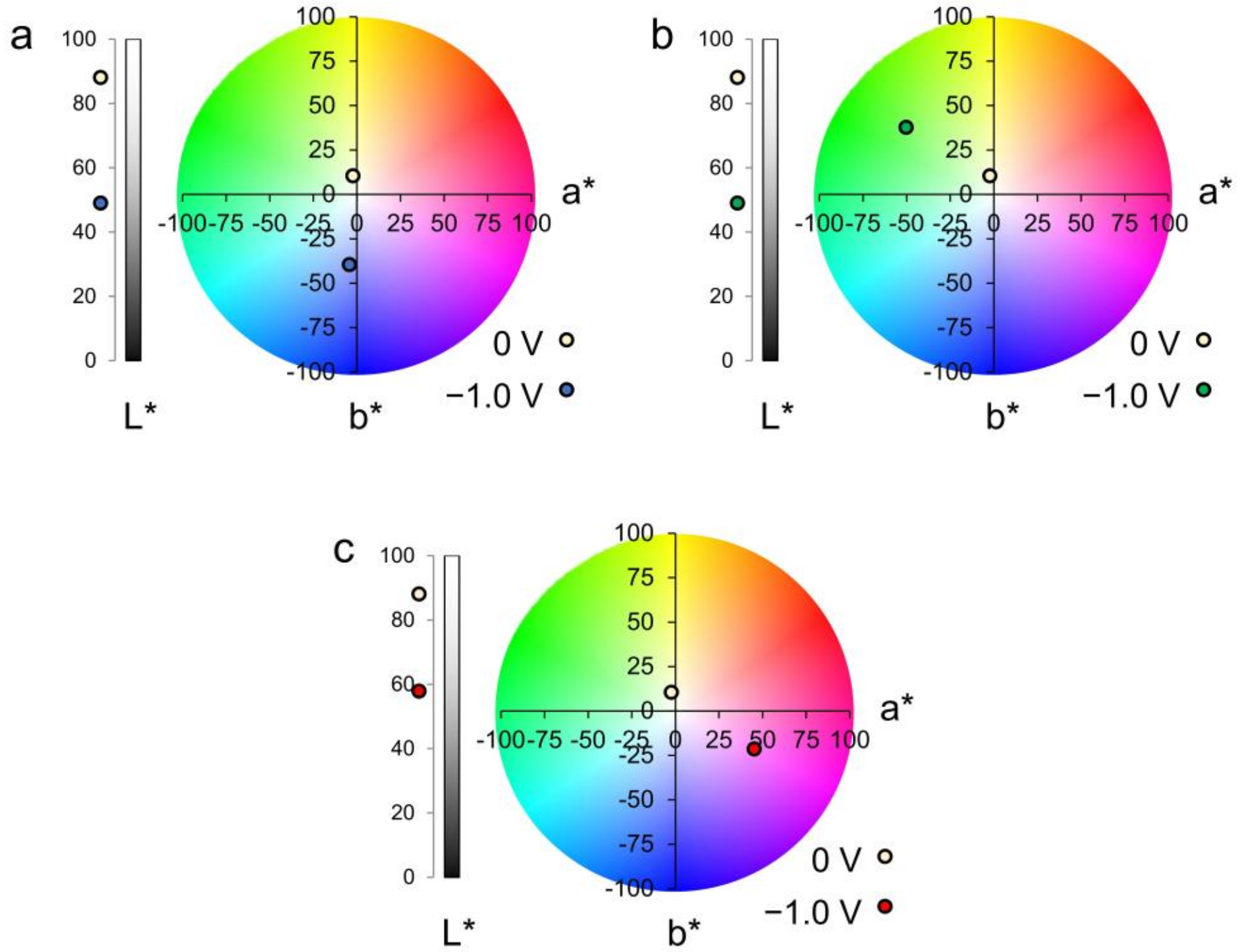

Figure S7. CIE 1976 Lab color coordinates of (a) MV-, (b) PV-, and (c) M-bisV-based EC devices at 0 V and −1.0 V. At 0 V color coordinates are [91.67, –0.89, 8.52], [90.41, –2.56, 13.08], and [90.59, –1.08, 9.61] for MV-, PV-, and M-bisV-based EC devices, respectively. At -1.0 V color coordinates are [54.15, –3.78, –38.29], [53.25, –49.69, 37.65], and [59.62, 45.84, –21.10] for MV-, PV-, and M-bisV-based EC devices, respectively.

a

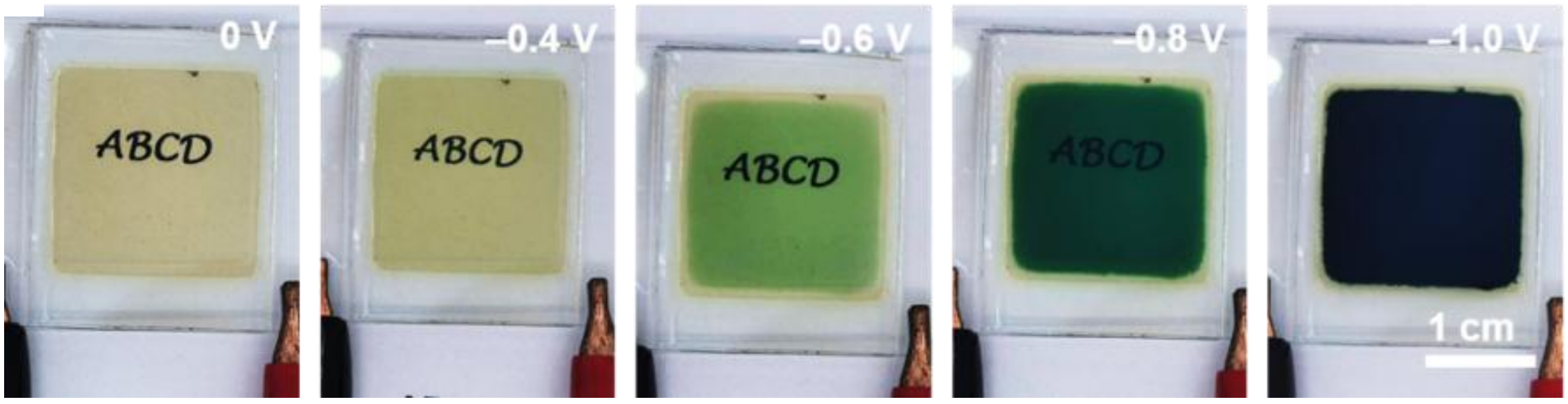

b

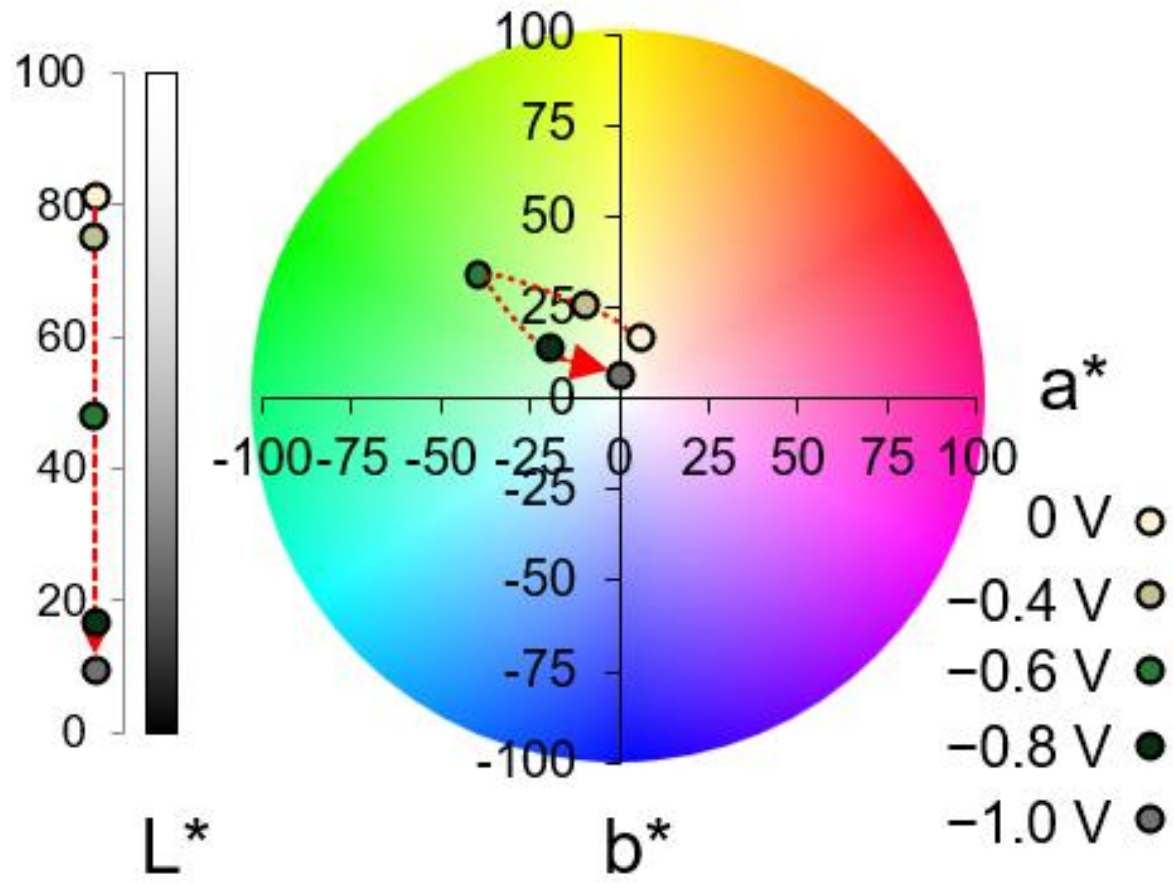

Figure S8. (a) Digital photographs of a TriV-based EC device recorded at different applied voltages (0, −0.4, −0.6, −0.8, and −1.0 V). (b) Evolution of the CIE 1976 Lab color coordinates of a TriV-based EC device when increasing the operating voltage from 0 to −1.0 V.

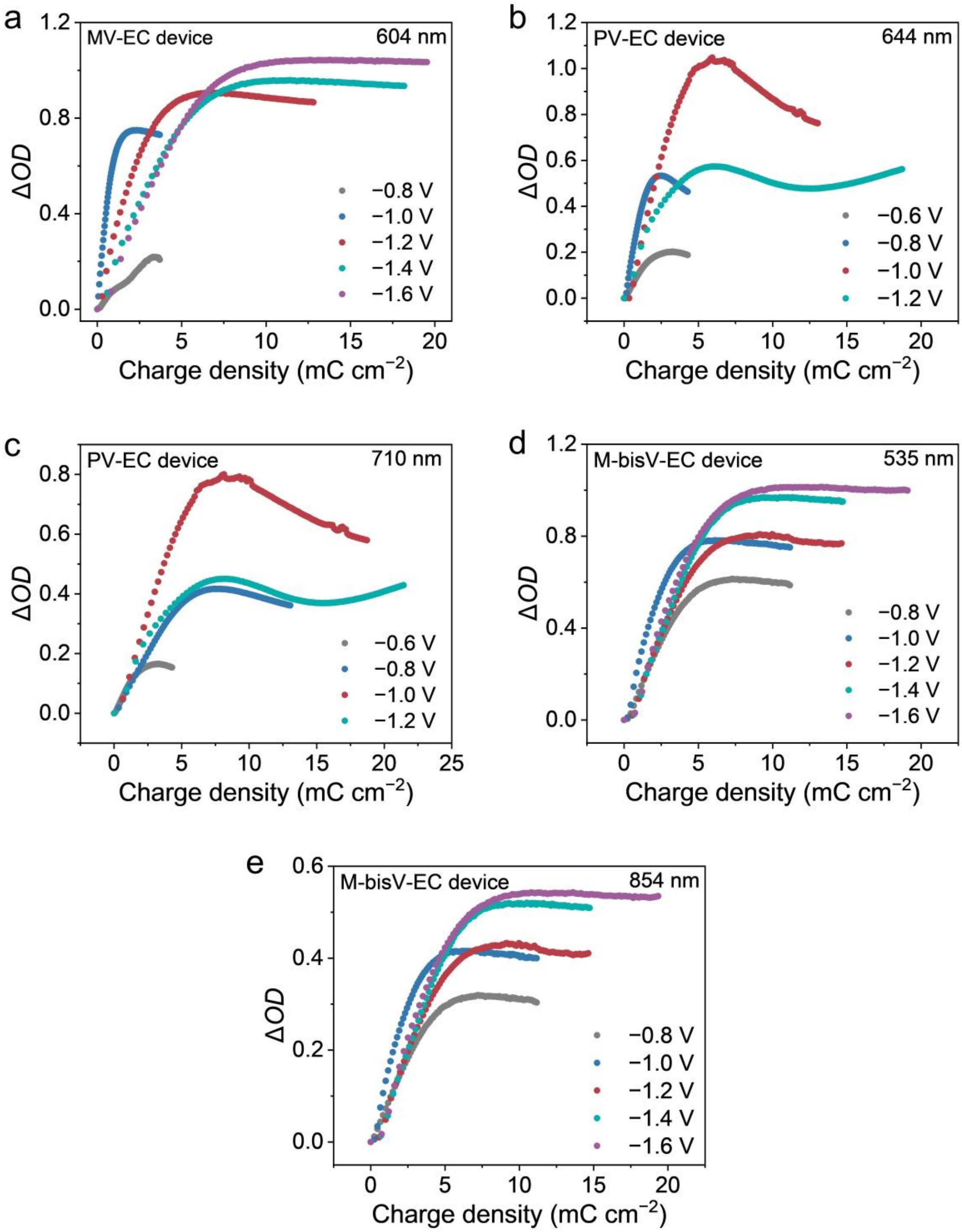

Figure S9. Optical density versus charge density for MV-, PV-, and M-bisV-based EC devices under different voltages.

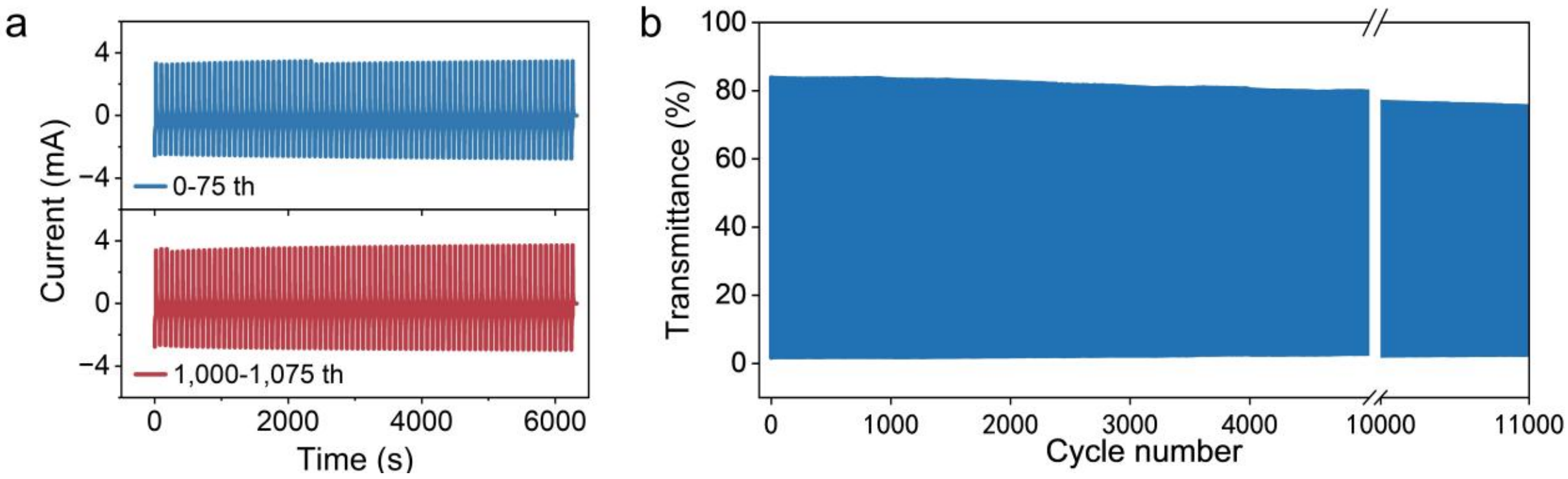

Figure S10. (a) Chronoamperometry curves of the TriV-based EC device for the first and last 75 cycles of the 1000 cycling test. (b) Variation of the transmittance at 604 nm between the bleached and colored states of the TriV-based EC device during an extended cycling test (11000 switching cycles at −1.0 V). At the end of the cycling test, the device preserved 89% of the initial transmittance modulation.

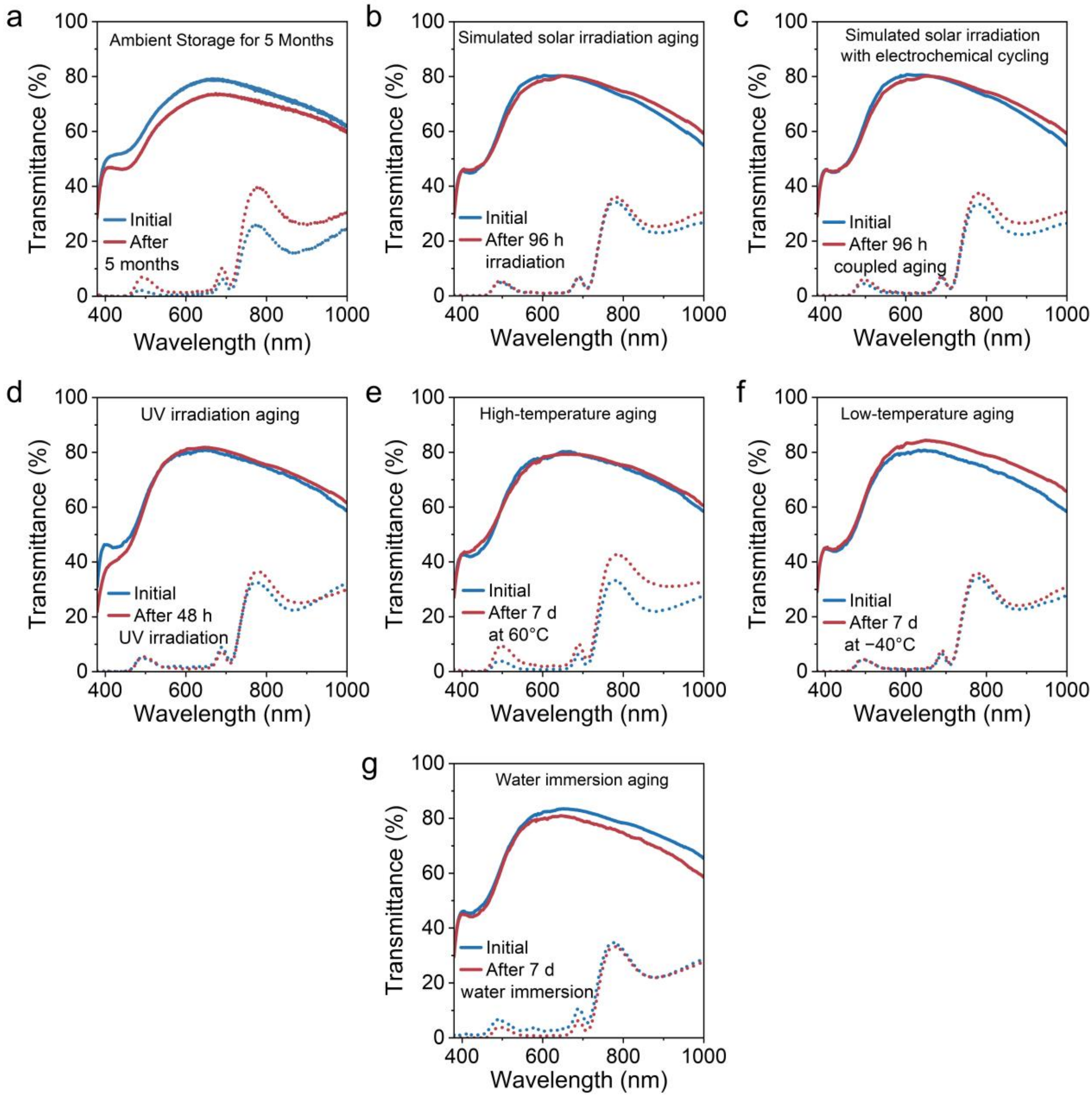

Figure S11. Transmittance spectra of the TriV-based EC device before and after the following aging experiments: (a) ambient storage for 5 months; (b) simulated solar irradiation (AM1.5G, 1000 W m^{-2}) for 96 h; (c) simulated solar irradiation for 96 h (AM1.5G, 1000 W m^{-2}) with periodic EC switching (100 electrochemical switching cycles every 12 h, with each cycle lasting 80 s); (d) 365 nm UV irradiation (133 W m^{-2}) for 48 h; (e) thermal aging at 60 °C for 7 days; (f) low-temperature aging at −40 °C for 7 days; and (g) water immersion for 7 days after sealing the edges of the device with a UV-curable adhesive (see Figure S12). Solid and dotted lines represent the bleached and colored states, respectively. Blue and red curves correspond to the initial and aged devices, respectively.

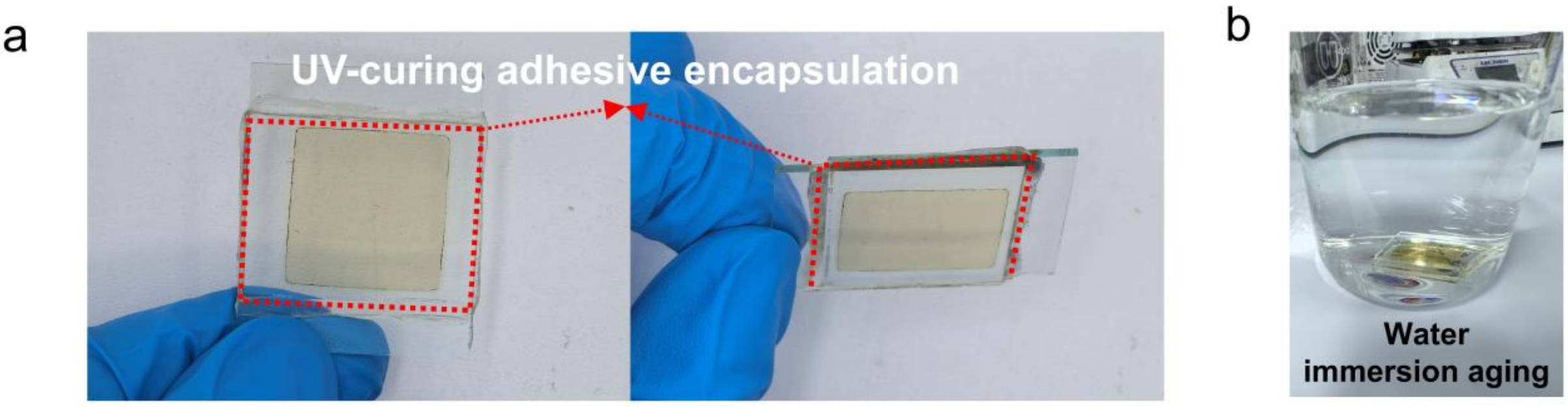

Figure S12. (a) Photograph of the edge-sealed TriV-based EC device using UV-curable adhesive. (b) Photograph of the edge-sealed EC device immersed in water.

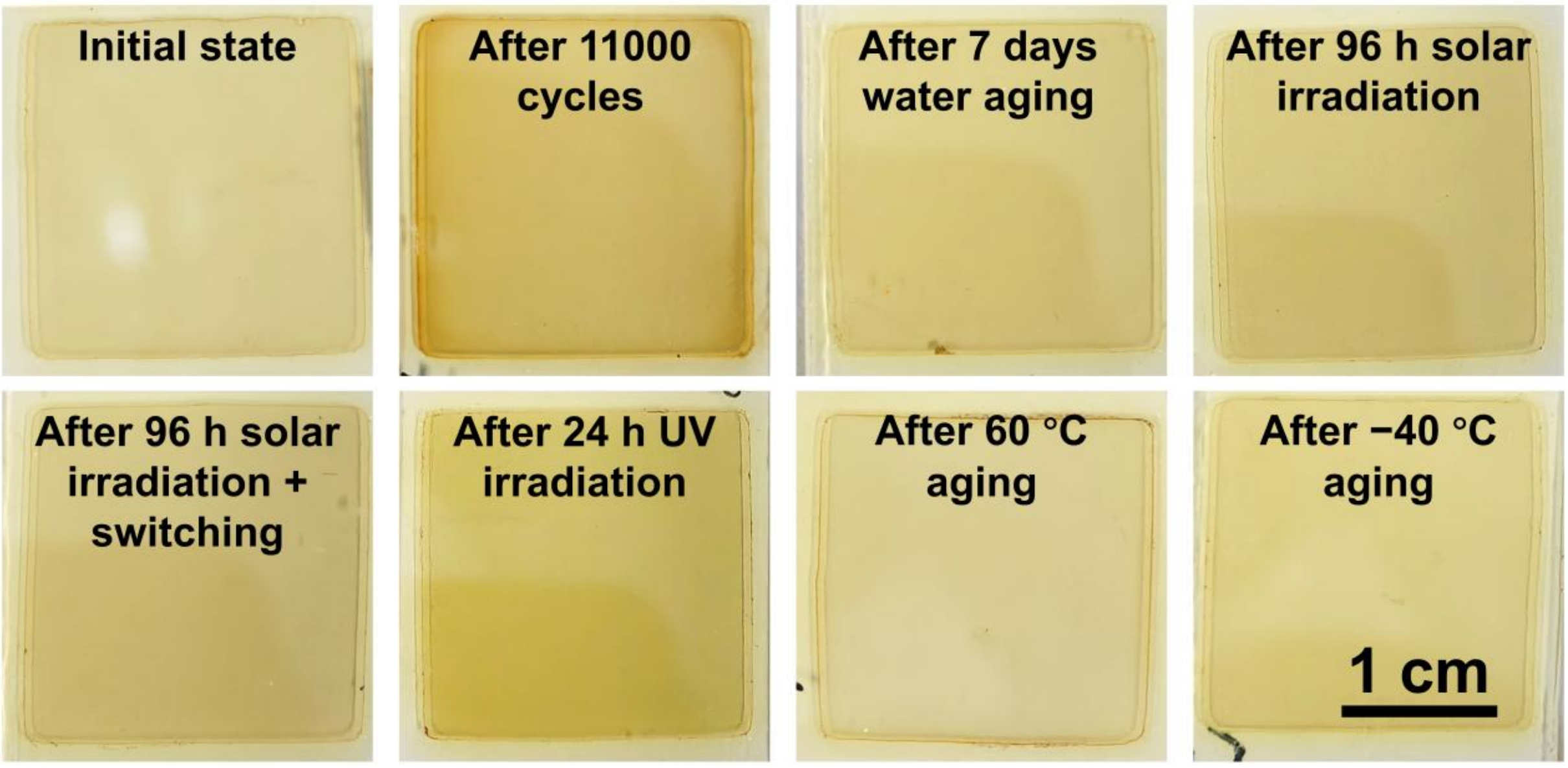

Figure S13. Photographs of the TriV-based EC device in its initial state and after 11000 switching cycles, 7 days of water immersion after edge sealing, 96 h solar simulator irradiation, 96 h solar simuator irradiation with periodic EC switching, 48 h UV irradiation, and thermal aging at 60 °C and −40 °C. No visible electrolyte leakage, bubble formation, or interfacial delamination was observed. Scale bar: 1 cm.

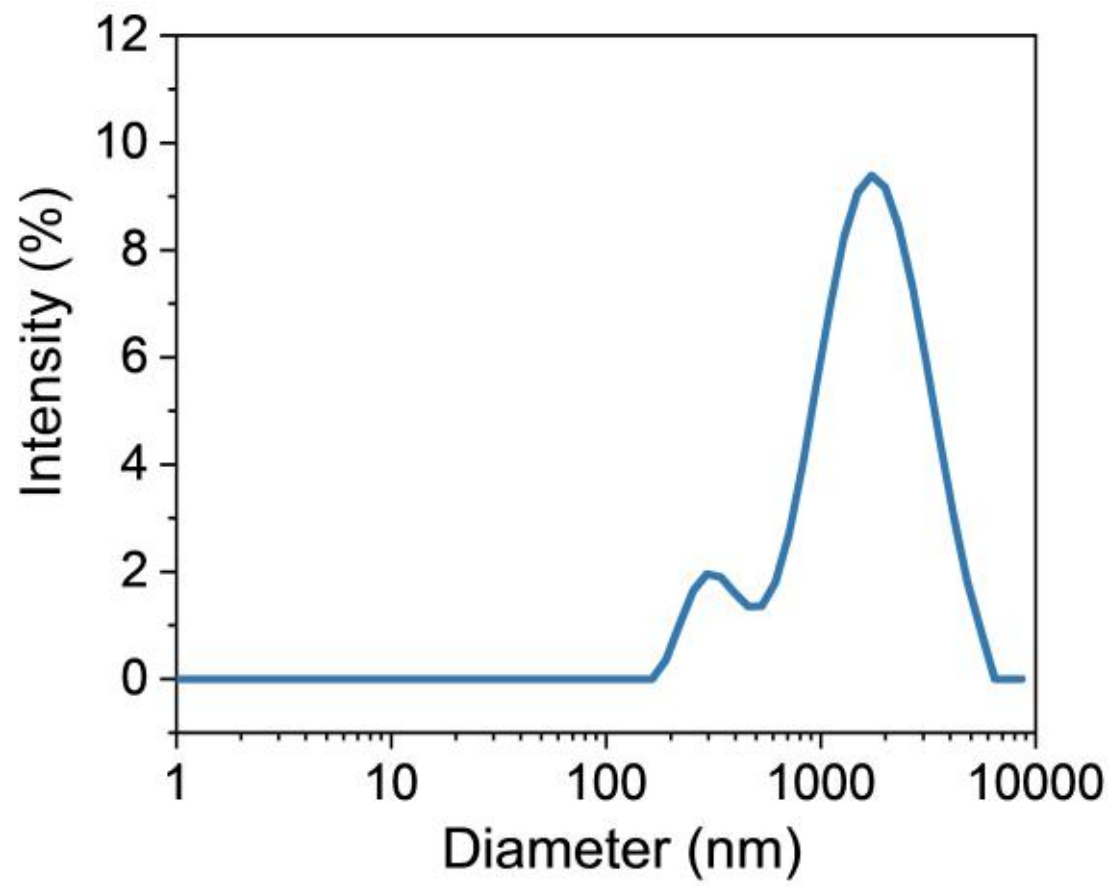

Figure S14. Dynamic light scattering profile of the aqueous paraffin-PVA emulsion used for the preparation of the TC layer of the hybrid ETC device.

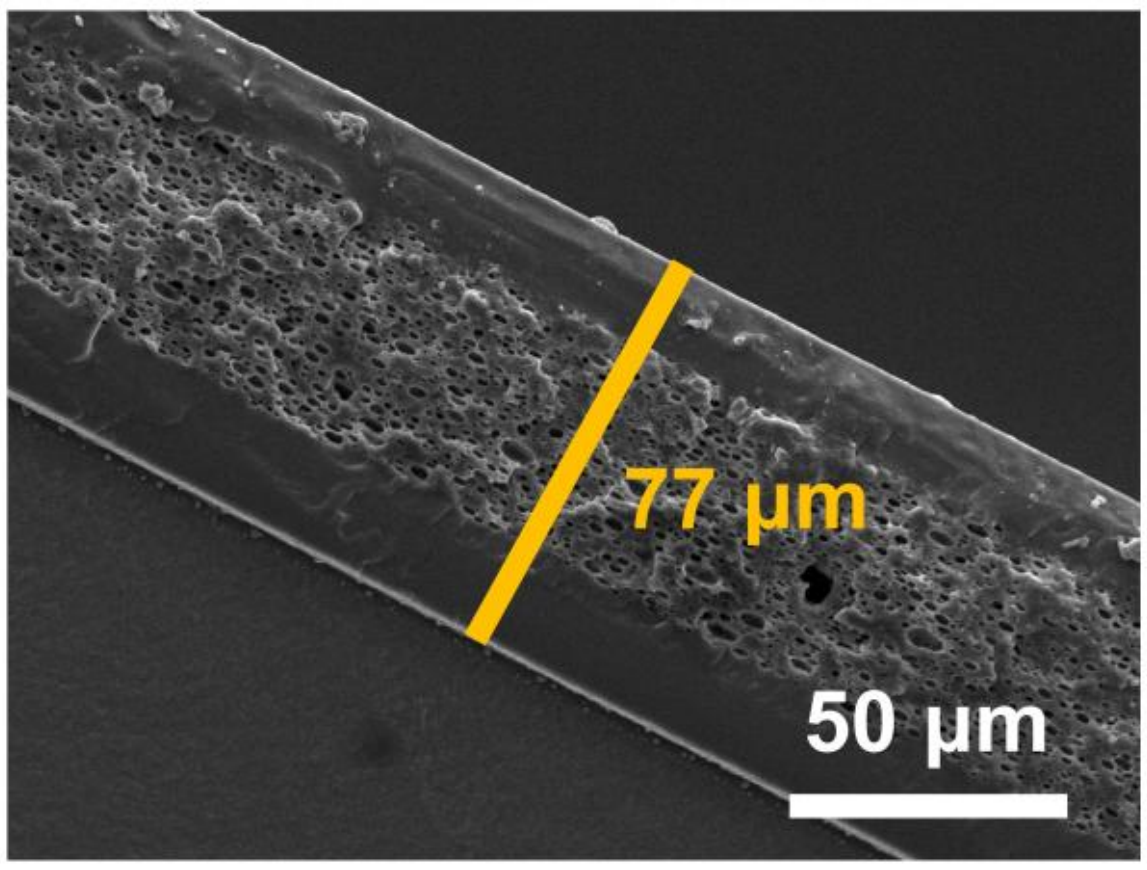

Figure S15. SEM image of the cross-section of a paraffin-based TC film composed of three consecutive layers of (1) PVA/glycerol, (2) PVA loaded with paraffin microparticles, and (3) PVA/glycerol. Before analysis, the film was treated with $CHCl_3$ for selective paraffin removal, thereby leaving visible voids in the central PVA layer.

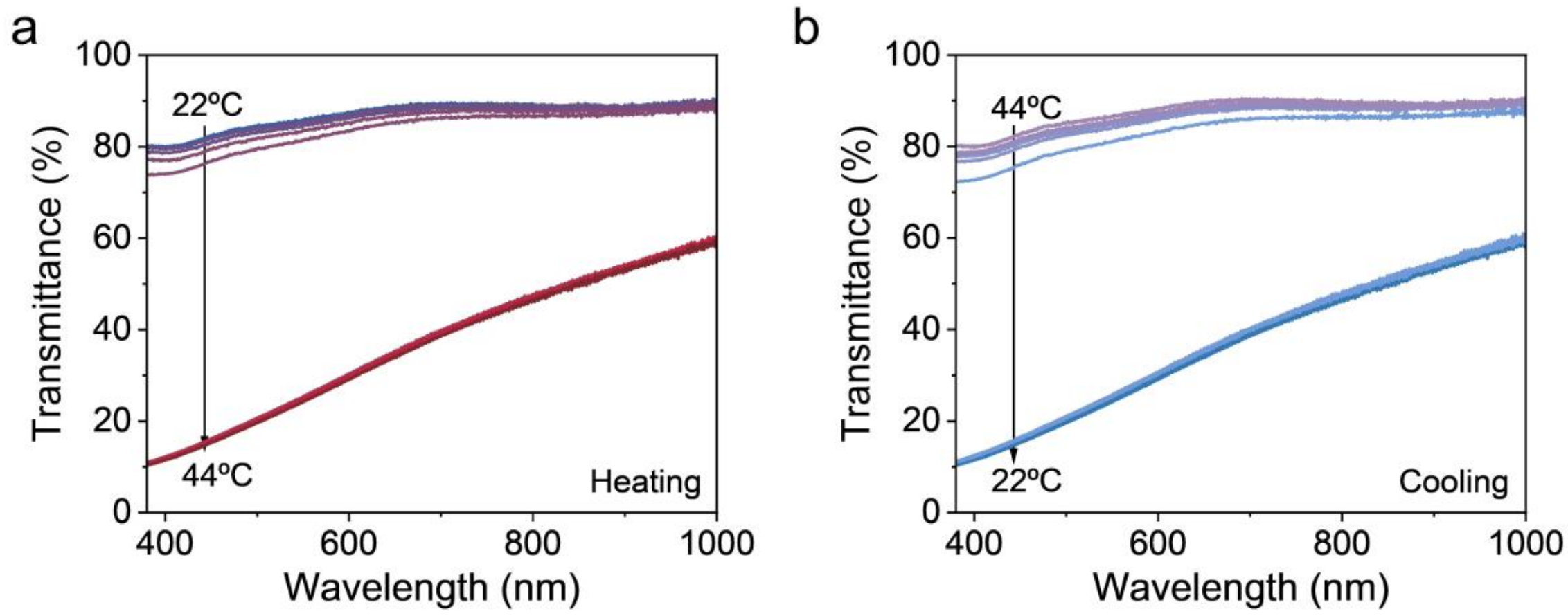

Figure S16. UV-vis transmittance spectra of a paraffin-based TC layer deposited on PET and subjected to (a) heating and (b) cooling processes with measurements taken every 2 °C.

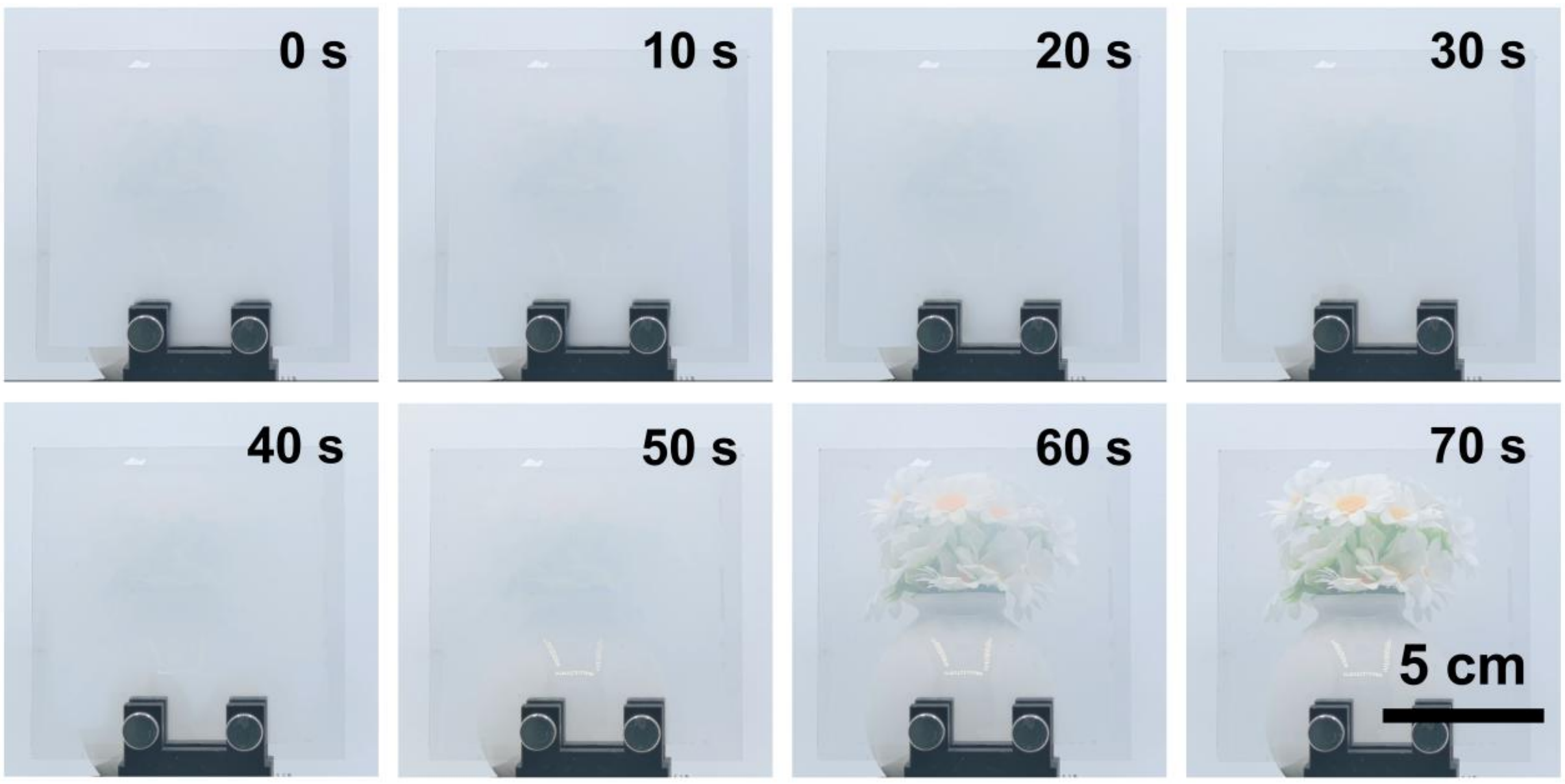

Figure S17. Digital photographs of the paraffin@PVA TC layer during recovery from the opaque state. The TC layer was first heated to 50 °C and then transferred to a 25 °C environment for natural cooling. Scale bar: 5 cm.

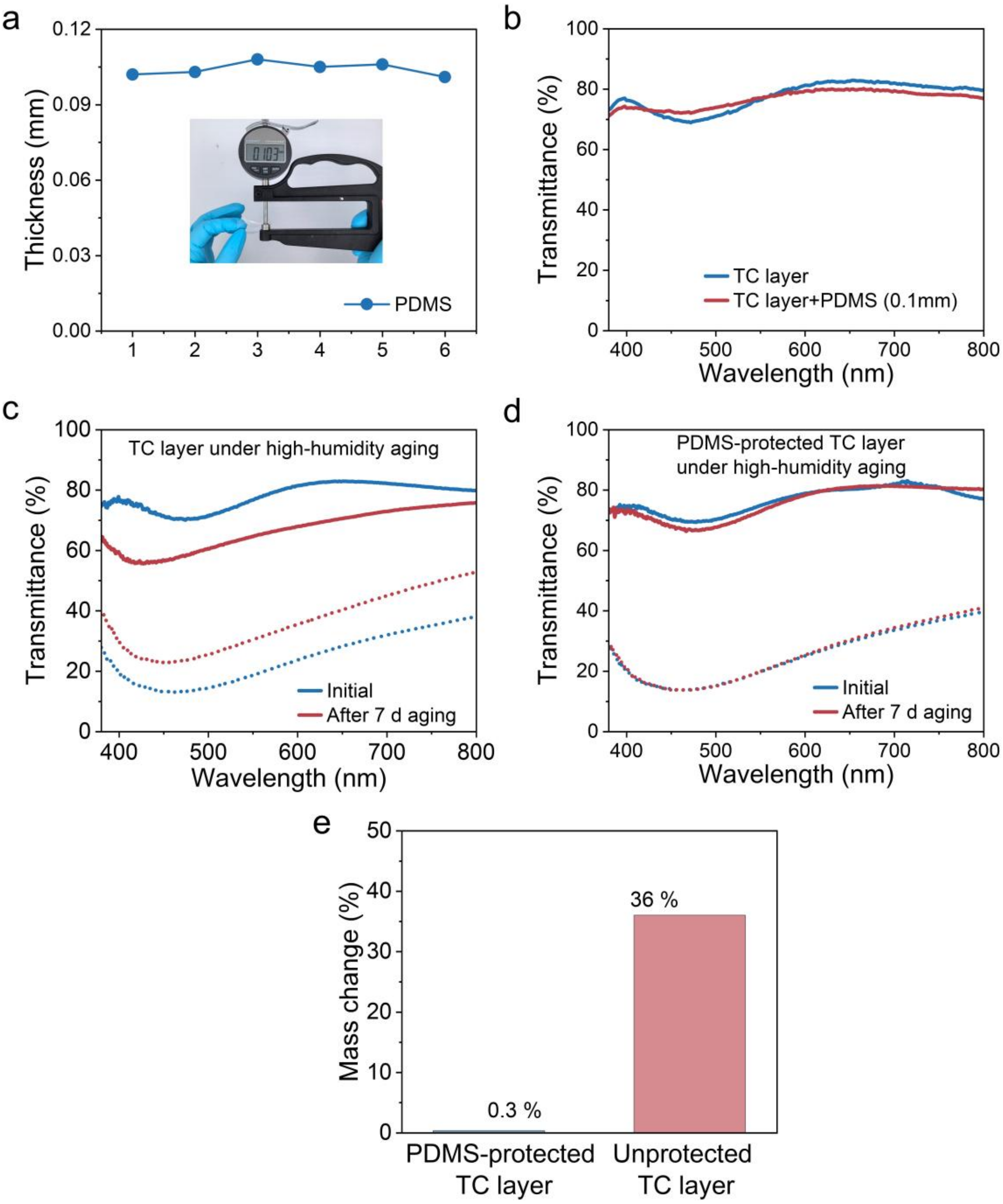

Figure S18. (a) Thickness distribution of the blade-coated PDMS protective layer over a paraffin@PVA TC layer. (b) Transmittance spectra of the TC layer before and after PDMS coating. (c) Transmittance spectra of the unprotected TC layer before and after humidity aging (85% RH). Spectral data is given at room temperature (solid lines) and after heating (dashed lines) to promote paraffin melting and transparent-to-opaque transition. (d) Transmittance spectra of the PDMS-protected TC layer before and after humidity aging (85% RH). Spectral data is given at room temperature (solid lines) and after heating (dashed lines) to promote paraffin melting and transparent-to-opaque transition. (e) Mass change of the unprotected TC layer and the PDMS-protected TC layer after humidity aging (85% RH for 7 days).

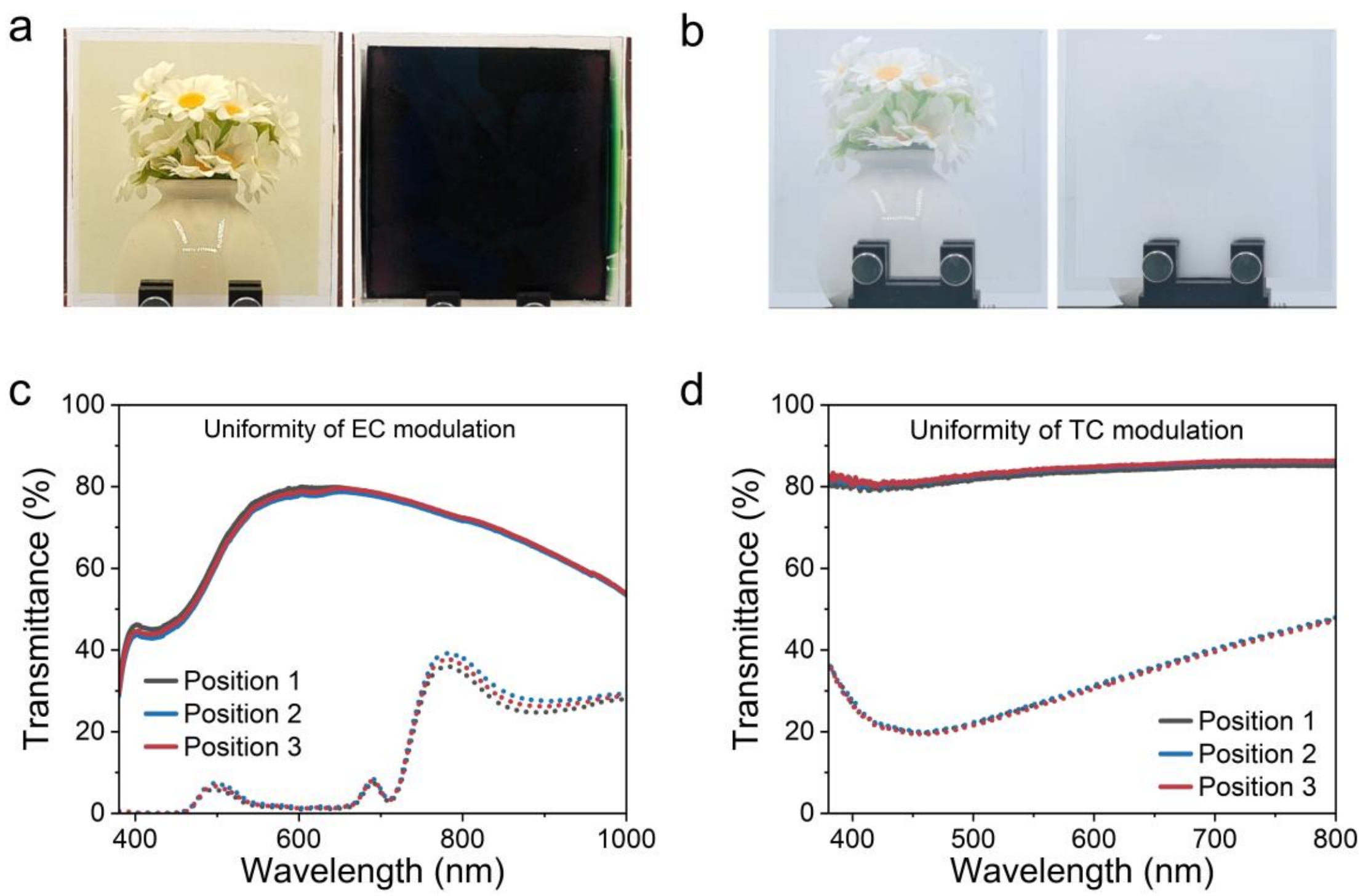

Figure S19. (a) Photographs of a 10 × 10 cm^2 TriV-based EC device in the bleached and colored states. (b) Photographs of a 10 × 10 cm^2 paraffin@PVA TC layer in the transparent and opaque states. (c-d) Position-dependent transmittance spectra of (c) the EC device and (d) the TC layer, which demonstrate good uniformity of their stimulus-responsive optical response. Solid and dotted lines represent the bleached and colored/opaque states, respectively.

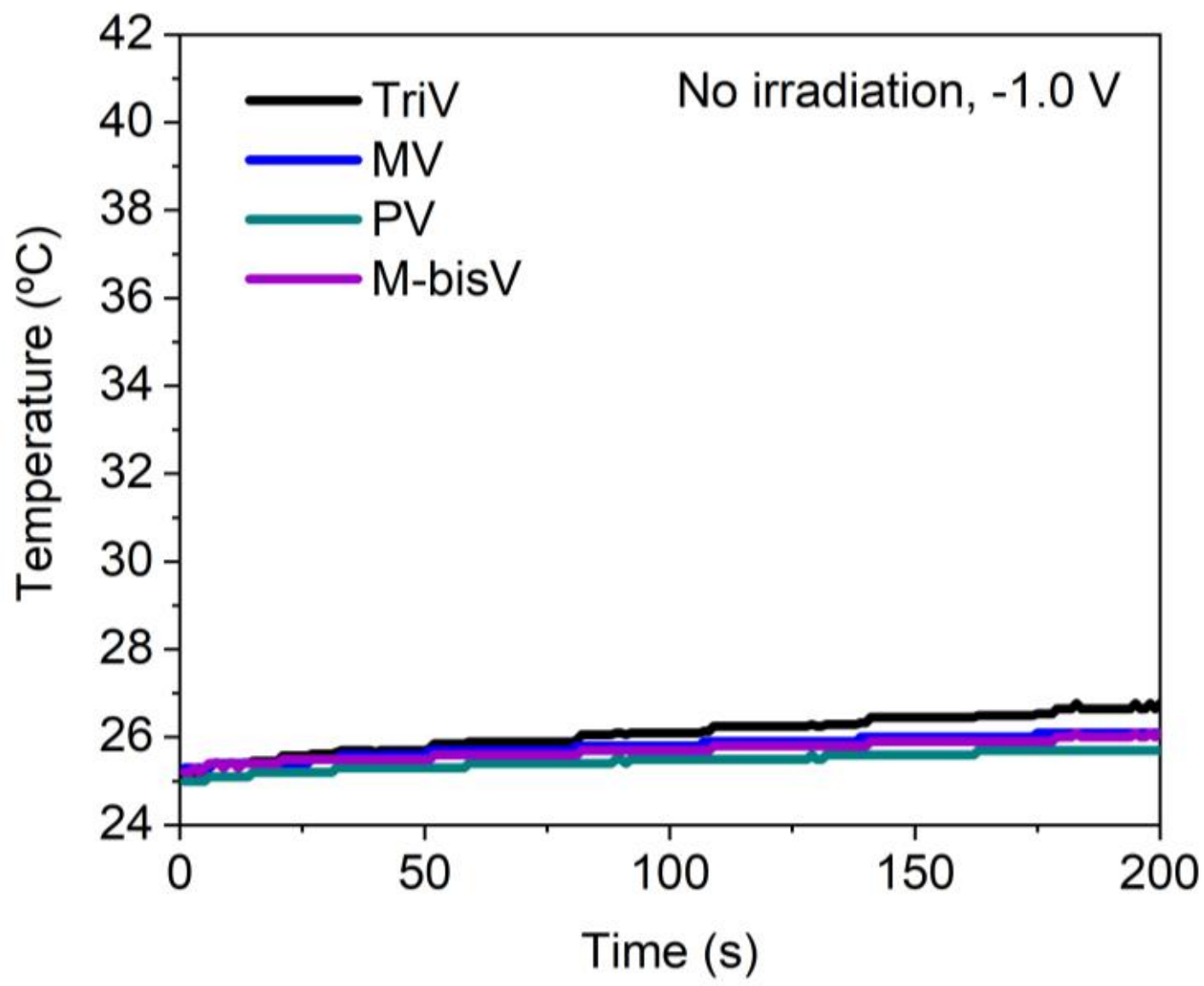

Figure S20. Surface temperature increment induced by the electrothermal heating caused upon operating TriV-, MV-, PV-, and M-bisV-based ETC devices at −1.0 V in the absence of solar irradiation. The external temperature was 25 ºC.

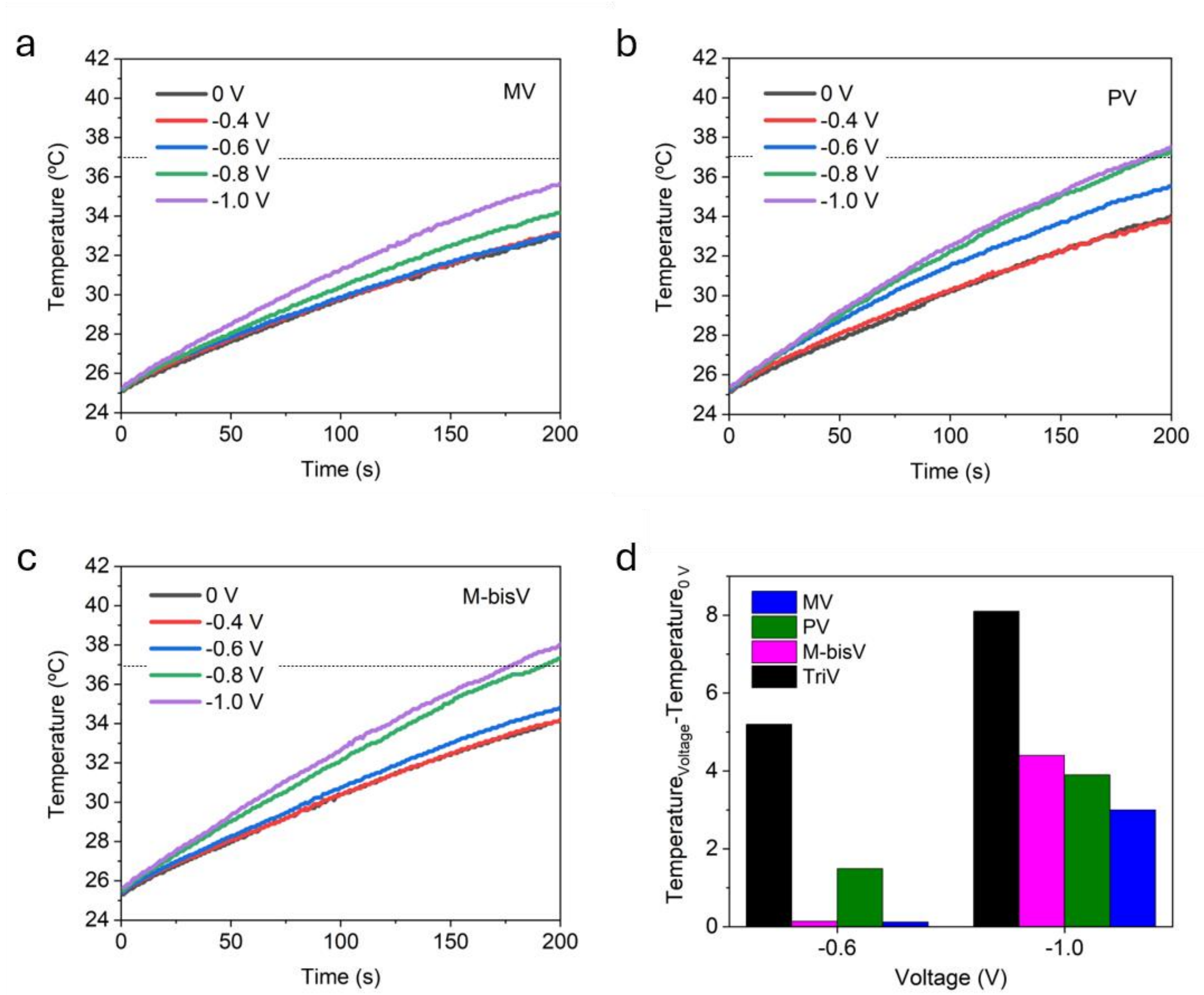

Figure S21. (a-c) Effect of the applied voltage on the temperature variations achieved at the surface of (a) MV-, (b) PV-, and (c) M-bisV-based ETC devices after 200 s of irradiation with a solar simulator (AM 1.5G, 1000 W m^{-2}). The dashed line indicates the melting temperature of the paraffin particles within the TC layer. (d) Difference in photothermally-assisted temperature achieved between the colored (−0.6 V or −1.0 V) and bleached (0 V) states of MV-, PV-, M-bisV- and TriV-based ETC devices after 200 s of irradiation with a solar simulator (AM 1.5G, 1000 W m^{-2}). In all experiments, the external temperature was 25 ºC.

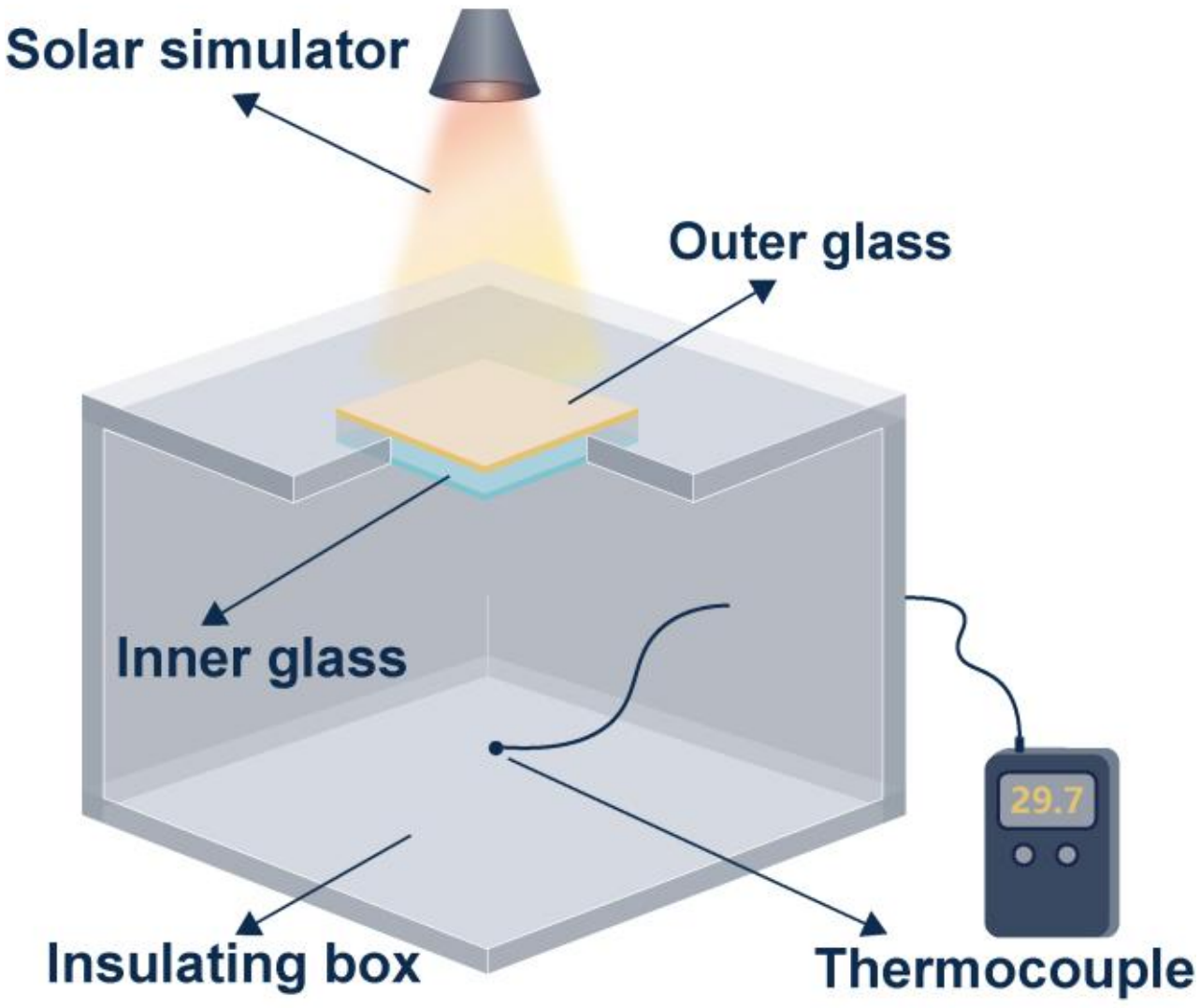

Figure S22. Schematic of the custom-built thermally insulated testing platform used for evaluating indoor heat regulation under simulated solar irradiation.

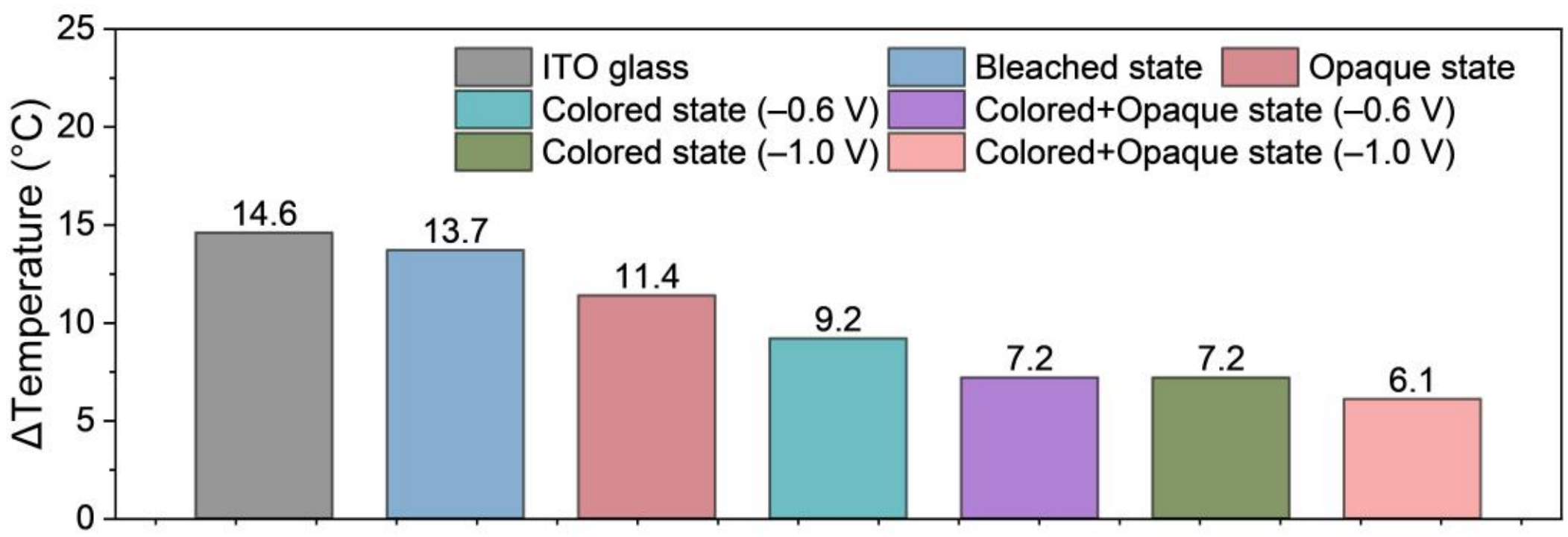

Figure S23. The temperature difference between the initial state and the steady activated state achieved inside an insulating box irradiated with a solar simulator (AM 1.5G, 1000 W m^{-2}) through a 3 × 3 cm^2 double pane window. Results are shown for different compositions of the inner pane and operation modes: ITO glass, EC layer operated at 0, –0.6 and –1.0 V (bleached and colored states), and a hybrid ETC window operated at 0, –0.6 and –1.0 V (opaque and colored+opaque states).

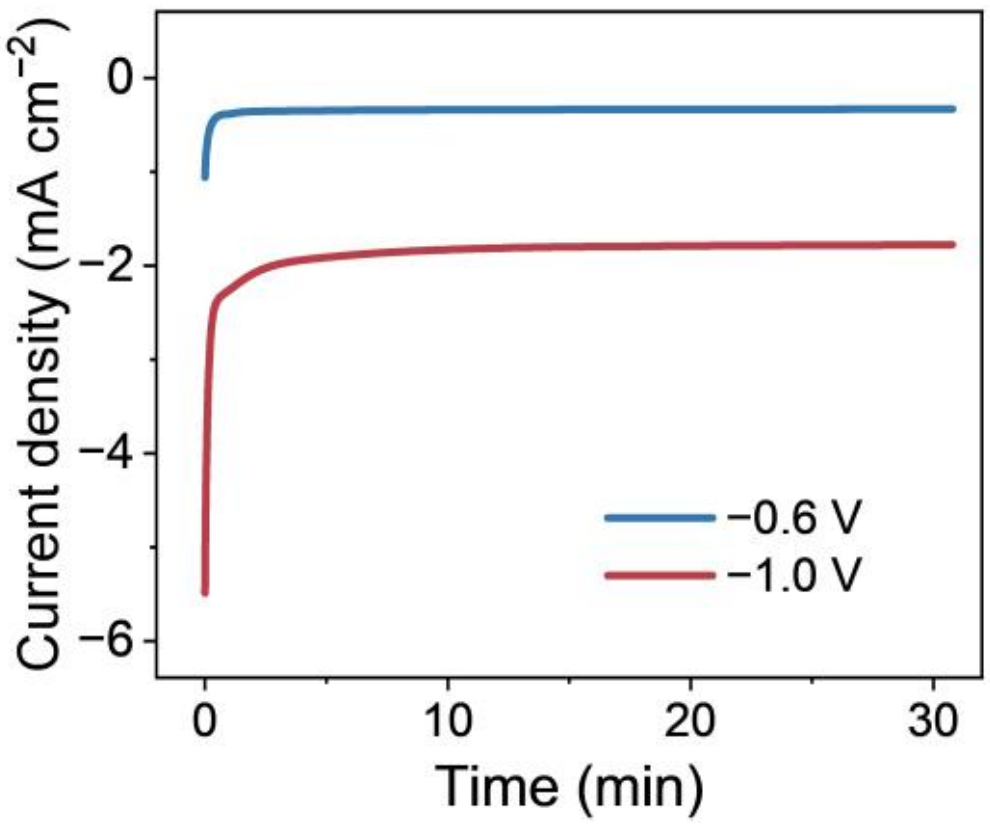

Figure S24. Chronoamperometry (CA) curves of TriV-based EC device under different voltages.

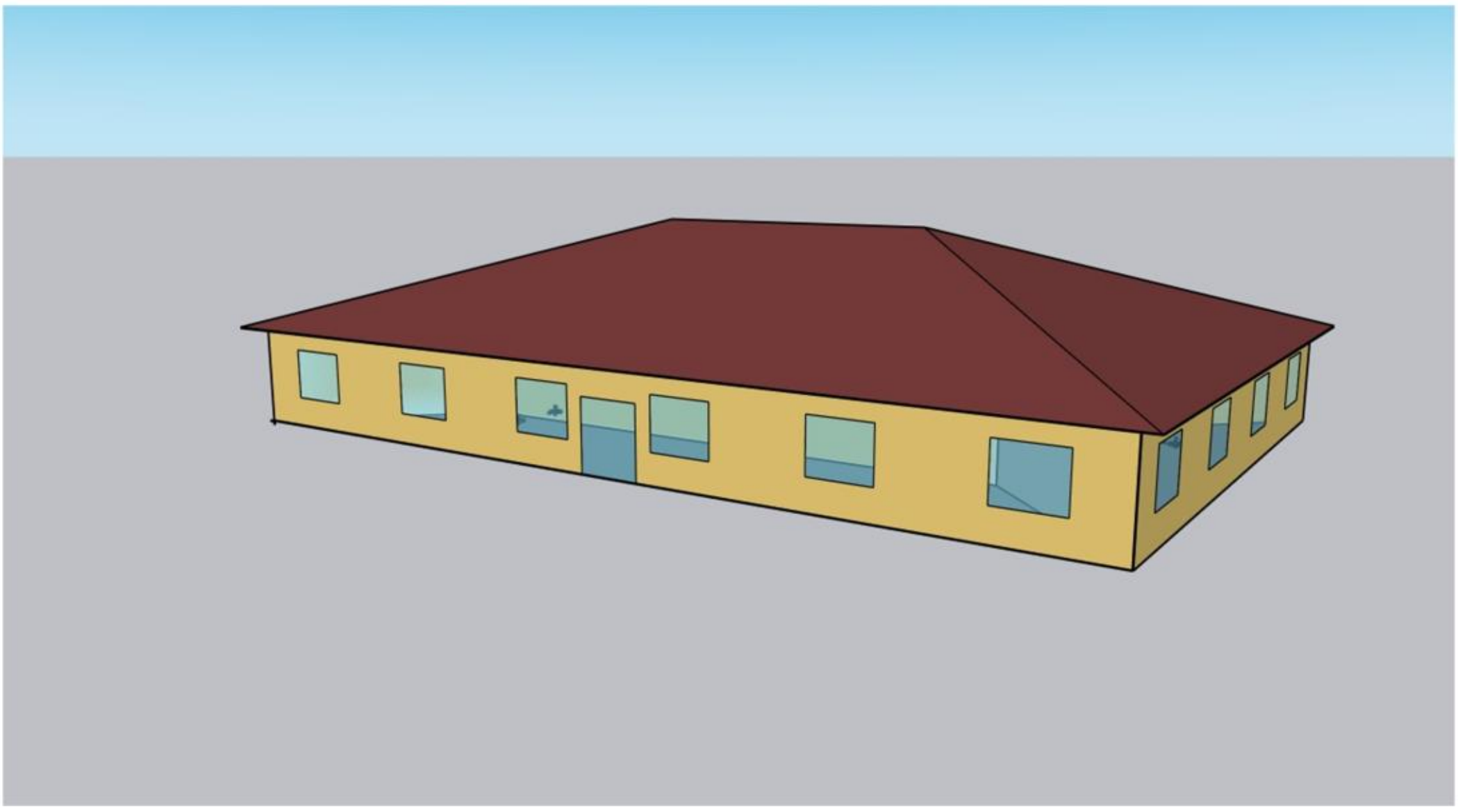

Figure S25. Building model used in energy simulations.

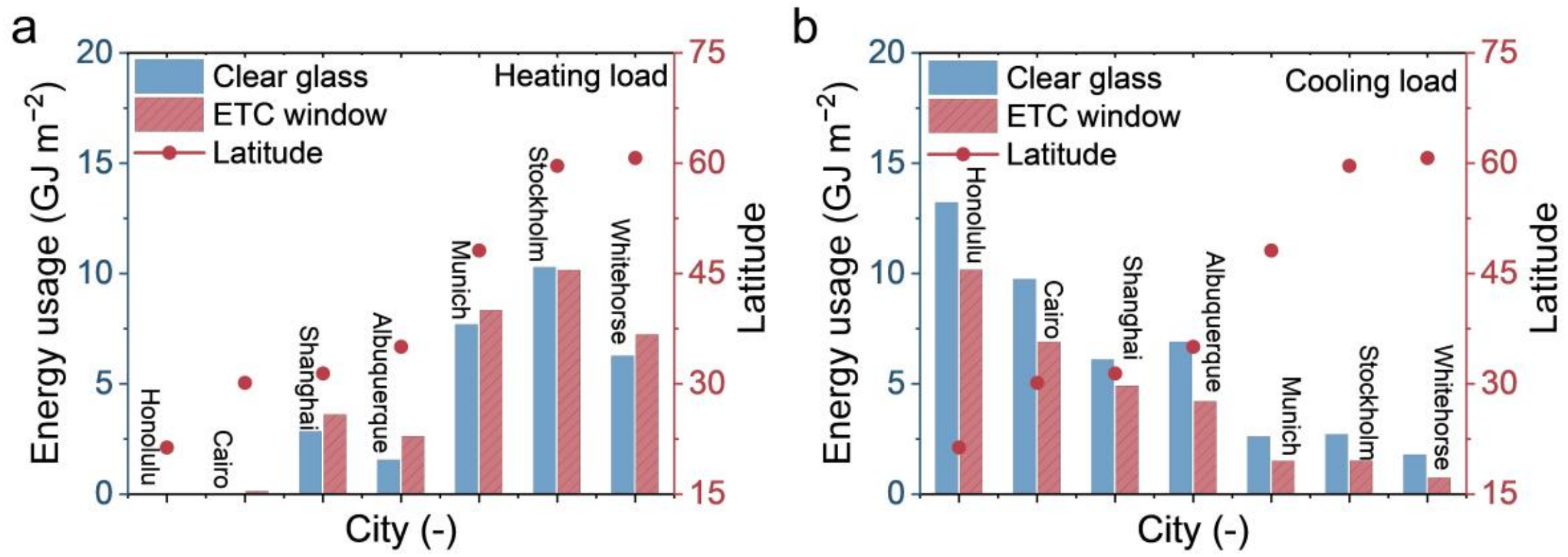

Figure S26. Simulated heating and cooling consumption of a full-scale building model in various cities, including Honolulu, Cairo, Shanghai, Albuquerque, Munich, Stockholm and Whitehorse, using normal glass and ETC smart window.

Table S1. Properties and parameters of MV, PV, and M-bisV in EC gels, including electron transfer number (n) for the reduction wave, concentration, and diffusion coefficient.

Material	n	Concentration (mmol L^{-1})	Diffusion coefficient (cm s^{-2})
MV	1	20	2.36×10^{-7}
PV	1	20	1.97×10^{-7}
M-bisV	2	10	8.82×10^{-8}

Table S2. Voltage-dependent CIE 1976 L*a*b* color coordinates and corresponding colors of a TriV-based EC device.

Voltage (V)	L*	a*	b*	
0	82.35	2.27	15.59	
−0.4	77.42	−8.43	25.47	
−0.6	46.37	−42.57	34.33	
−0.8	17.45	−22.12	13.81	
−1.0	13.16	0.33	3.54	

Table S3. Coloration efficiency (CE, cm^2 C^{-1}) of MV, PV, and M-bisV-based EC devices measured at different applied voltages.

Voltage	MV (604 nm)	PV (644 nm)	PV (710 nm)	M-bisV (535 nm)	M-bisV (854 nm)
−0.6		113	93		
−0.8	90	108	80	145	74
−1.0	182	185	140	249	145
−1.2	167	101	94	172	97
−1.4	154			178	93
−1.6	150			189	100

Table S4. Comparison of the performance of our TriV-based EC device with other broadband electrochromic smart windows in the literature.

Ref	EC material	$T_{bleached}$ [a]	ΔT [b]	Operating voltage (V) [c]	t_c/t_b (s) [d]	Cycling durability [e]	Device area (cm^2)	Colored state [f]	Temperature decrement [g]
This work	MV/PV/ M-bisV	65%	70% at 604 nm 53% at 854 nm	−1.0/0	7.4-15.7/ 8.8-20.2	89% (11000 cycles)	100	Black	6.5 ºC
[1]	$EV^{2+}{}_2$/TPD/ H_2QSK	99%	64% at 500 nm 38% at 1100 nm	1.4/0	4.4/4.9	94% (15000 cycles)	100	Black	5.8 °C
[2]	NiCo-LDH/ECP	75%	66% at 472 nm 45% at 800 nm	0.8/−0.3	6.5/14.6	80% (2000 cycles)	100	Black cherry	-
[3]	FBV-Et viologen device	99%	99% at 670 nm 90% at 1425 nm	2.0/−0.5	4.5/4.6	100% (200 cycles)	6.25	Black	7.4 °C
[4]	V_2O_5/ITO dual-band ECD	34%	~5% at 550 nm 53% at 850 nm	−1.5/1.5	22/3.0	99% (10000 cycles)	-	Green	7.5 ºC
[5]	Zn electrodeposition	70%	73% at 600 nm 31% at 875 nm	−1.0/1.0	4/8	90% (1000 cycles)	625	Black mirror	8.4 °C
[6]	CuZn-RMED/PAM hydrogel	77%	57% at 550 nm 21% at 800 nm	−1.2/0.8	15/24	90% (3000 cycles)	400	Black	6-14 °C
[7]	n-PBDF/m-ITO	43%	41% at 550 nm 65% at 750 nm	−1.0/1.8	0.37/0.43	97% (27000 cycles)	4800	Black	-
[8]	WO_3/PVTC-QSPE/NiO	58%	48% at 633 nm 40% at 1000 nm	−1.4/1.4	4.8/3.2	86% (40000 cycles)	100	Black	-
[9]	PA-PANI/ Zn mesh	70%	50% at 530 nm 22% at 1000 nm	0-3.5	19.2/61.4	63-71% (10000 cycles)	25	Black	-

[10]	$TiNb_2O_7$	75%	65% at 600 nm<br>70% at 1200 nm	−3.8/2.0	23.4/12.4	78%<br>(500 cycles)	200	Black	5.0 ºC
[11]	m-WO_{3-x} nanowire	95%	64% at 700 nm<br>78% at 1600 nm	−1.3/1.0	-	-	-	Dark blue	6.6 °C

[a] Transmittance at 550 nm in the bleached state. [b] Transmittance modulation under operation for selected visible and NIR wavelengths. [c] Operation voltages for the coloration and bleaching processes. [d] Characteristic times of the coloration and bleaching processes. [e] Transmittance modulation retention (%) after the indicated number of switching cycles. [f] Color of the colored state. [g] Temperature decrement achieved indoors upon EC operation in energy efficiency experiments.

Table S5. Comparison of the performance of our paraffin@PVA TC layer with other thermochromic smart windows in the literature.

**Ref**	**TC material**	**$T_{lum,cold}$** [a]	**ΔT_{lum}** [b]	**ΔT_{solar}** [c]	**$T_{switching}$ (ºC)** [d]	**Response time (s)** [e]	**Area (cm^2)**	**Fabrication** [f]	**Temperature decrement** [g]
This work	Paraffin@PVA[h]	66%	55%	39%	37/34	< 70	100	Blade coating	2.3 ºC
[12]	Paraffin@PVA[h]	77%	73%	59%	37/34	20	400	Blade coating	3.8 ºC
[13]	Paraffin@acrylate	78%	43%	38%	37/34	105	100	UV curing	4.4 ºC
[14]	Hydrated ionic polymer	88%	56%	30%	25-42	347/792	1200	Blade coating	<10 ºC
[15]	Ionogel	97%	89%	78%	28	300	2400	Solution casting	8.2 ºC

[16]	Ionogel	95%	90%	83%	27-30	300	400	Solution casting	8.8 ºC
[17]	Hydrogel + $Cs_{0.33}WO_3$	70%	70%	70%	18.9-28.9	~16	-	UV curing + $Cs_{0.33}WO_3$ coating	3.2 ºC
[18]	PCM@resin	90%	90%	86%	~47.6	-	-	UV-curing	-
[19]	VO_2	43%	4%	11%	-	-	4	Nanoimprinting + spin coating	4.0 ºC
[20]	Hydrogel	-	95%	76%	19.5/50.4	70/120	-	Sandwich filling	7.6 ºC
[21]	Ionogel	91%	-	79%	27-130 ºC	-	600	Roll-to-roll coating	6-8 ºC
[22]	PVP-NaBr solution	~65%	-	47%	10-70 ºC	-	10000	Liquid filling	7 ºC
[23]	Pereovskites	94%	58%	39%	34.3	72/306	25	Spin coating + vapor filling	7.9 ºC

[a] Transmittance in visible region in the transparent state. [b] Transmittance modulation under operation along the visible region. [c] Transmittance modulation under operation along the solar spectrum. [d] Switching temperatures, which can be tuned for some TC materials. [e] Response time for the opaque-to-transparent transition upon cooling. [f] Fabrication processes. [g] Temperature decrement achieved indoors upon TC operation in energy efficiency experiments. [h] Equivalent TC layers displaying distinct thermochromic responses. The system described in ref. [12] is a stand-alone TC layer exhibiting enhanced thermo-optical performance. In this work, the same layer was coupled to a Tri-V-based EC device, whose non-negligible absorption and scattering of sunlight in its bleached state reduced the initial transparency and transmittance modulation of the paraffin@PVA layer.

Table S6. Preliminary raw-material cost estimation of the ETC smart-window device.[a]

**Device part**	**Material / item**	**Unit price**	**Amount used for 1 m^2 device**	**Cost contribution**
Conductive substrate	ITO conductive glass	35.00 € m^{-2}	2 sheets m^{-2}	70.00 € m^{-2}
EC gel	Propylene carbonate	29.75 € L^{-1}	0.2 L m^{-2}	5.95 € m^{-2}
	Ferrocene	0.74 € g^{-1}	2.232 g m^{-2}	1.72 € m^{-2}
	[EMIM][TFSI]	4.28 € g^{-1}	15.65 g m^{-2}	66.98 € m^{-2}
	Poly(methyl methacrylate)	0.29 € g^{-1}	120 g m^{-2}	34.80 € m^{-2}
	Methyl viologen	41.18 € g^{-1}	2.98 g m^{-2}	122.71 € m^{-2}
	Phenyl viologen[b]	45.05 € g^{-1}	1.92 g m^{-2}	86.50 € m^{-2}
	Methyl-bridged bisviologen[b]	45.99 € g^{-1}	3.48 g m^{-2}	160.02 € m^{-2}
TC layer	PVA/glycerol layer	6.40 € m^{-2} per layer	2 layers	12.80 € m^{-2}
	Paraffin emulsion layer	8.93 € m^{-2} per layer	1 layer	8.93 € m^{-2}
Total	ETC device raw-material cost		1 m^2	570.41 € m^{-2}

[a] Cost estimation from raw materials purchased from chemical distributors, without considering other factors such as processing and labor costs. [b] Estimated from the cost of the raw materials for their synthesis using reported procedures and yields: 4,4'-bipirydine, 2,4-dinitrochlorobenzene, aniline and ammonium hexafluorophosphate for PV; and 4,4'-bipyridine, 1,3-diiodopropane, iodomethane and ammonium hexafluorophosphate for M-bisV.

Table S7. Comparison of the performance of our hybrid ETC device with other combined electro-thermochromic smart windows in the literature.

Ref	TC material	EC material	$T_{lum}^{bleached}$ [a]	$\Delta T_{solar}^{hot,EC}$ [b]	$T_{switching}$ (ºC) [c]	EC color [d]	EC operation voltage (V) [e]	Synergistic effects [f]	Temperature decrement [g]
This work	Paraffin@PVA	Viologen mixture	66% (0 V)	50%	37/34	Black	−1.0/0	Yes (−1.0 V)	7.5 ºC
[24]	Hydrogel	Conjugated polymers	78% (−1.5 V)	58%	31.5	Blue	+1.5/−1.5	Yes (1.5 V)	11.8 ºC
[25]	W/VO_2	Liquid crystal	69% (30 V)	-	-	White	0/30	No	12.0 ºC
[26]	Hydrogel	Viologen and prussian blue	-	-	30-35	Blue	−2.5/2.5	No	-
[27]	Hydrogel	Viologen	82% (0 V)	71%	33	Blue	−1.8/0	No	6.3 ºC
[28]	Hydrogel	WO_3-Prussian Blue	~70% (2.5 V)	52%	25-80 ºC	Blue	−1.0/2.5	No	-
[29]	Hydrogel	Extended viologen	~70% (−1.0 V)	-	33-35	Red	0/−1.0	No	5.0 ºC
[30]	Hydrogel	WO_3-NiO	~80% (2.5 V)	-	32	Blue	−2.5/2.5	No	~14.0 ºC

[a] Transmittance in visible region in the bleached state. The voltage needed to reach this high-transparency state is given in parentheses. [b] Transmittance modulation under combined EC and TC operation along the solar spectrum. [c] Switching temperature of the TC component. [d] Color of the electrochemically-induced colored state. [e] Voltages required for the coloration and bleaching processes of the EC component. [f] Synergistic effects due to photothermally-assisted activation of the TC component through sunlight absorption by the EC module. The optimal potential required to promote these effects is given in parentheses.

Table S8. Comparison of annual energy loads between ETC smart windows and clear glass across representative global cities.

Zone	Latitude	Cooling load (GJ)		Heating load (GJ)		Heating/cooling load (GJ)	
		**ETC window**	**Clear glass**	**ETC window**	**Clear glass**	**ETC window**	**Clear glass**
Honolulu, HI, U.S.	21.32	10.16	13.21	0	0	10.16	13.21
Cairo, Egypt	30.13	6.88	9.74	0.11	0	6.99	9.74
Shanghai, China	31.4	4.89	6.08	3.59	2.83	8.48	8.91
Albuquerque, NM, U.S.	35.04	4.19	6.89	2.6	1.53	6.79	8.42
Munich, Germany	48.13	1.48	2.59	8.32	7.68	9.8	10.27
Stockholm, Sweden	59.65	1.49	2.7	10.14	10.27	11.63	12.97
Whitehorse, YT, Canada	60.72	0.72	1.77	7.22	6.26	7.94	8.03

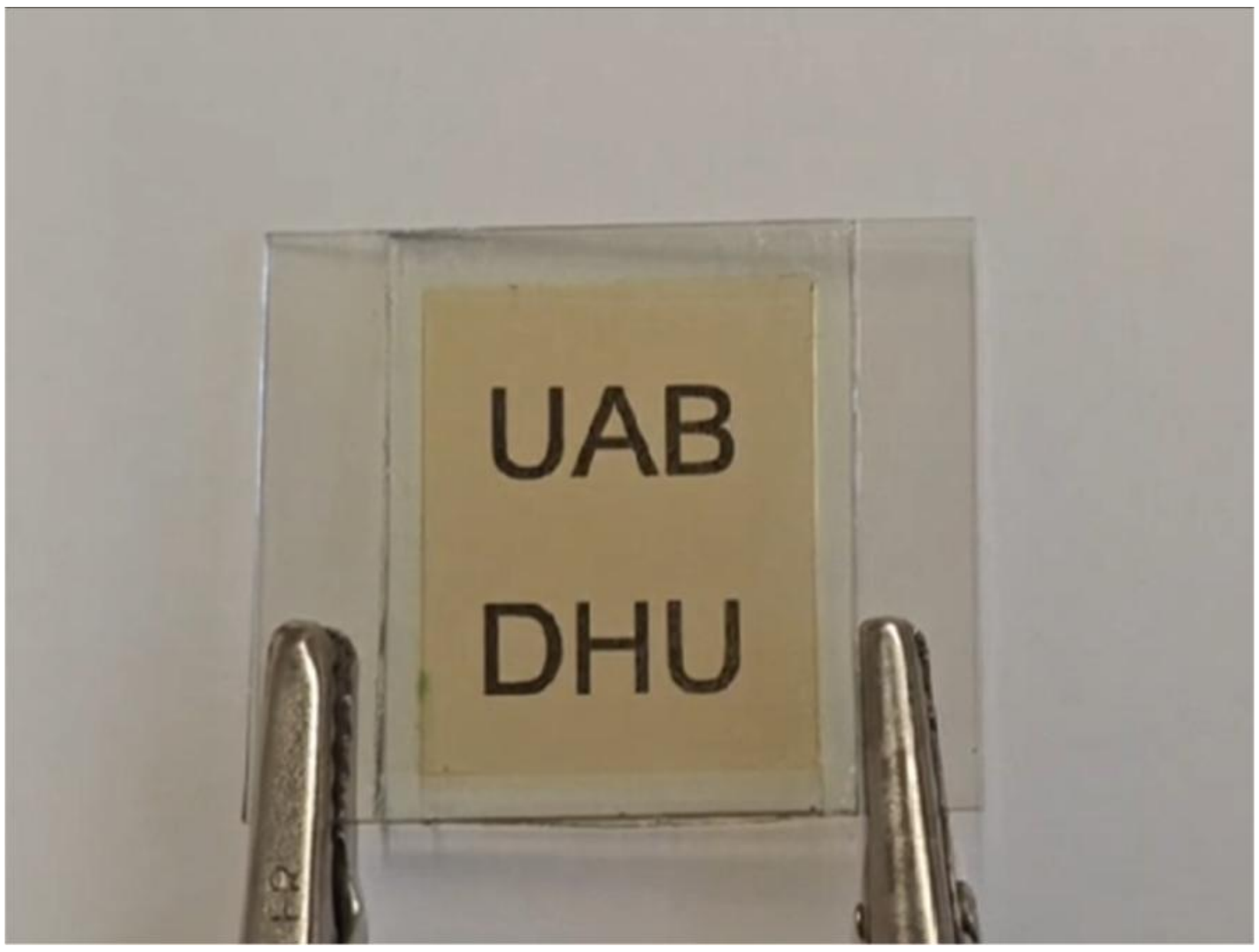

Movie S1. Real-time video of the coloration and color bleaching processes of a TriV-based EC gel sandwiched between ITO-glass substrates at -1.0 V and 0 V, respectively.

References

[1] X. Lv, R. Chen, R. Cheng, et al., "Enhanced cycling stability of black electrochromic device with hydroquinonesulfonate salt as an efficient redox mediator for energy-saving windows," *Chem. Eng. J.* **2026**, 533, 174588.

[2] Y. Gao, X. Li, Q. Ding, et al., "Robust photothermal regulation in large-area smart windows enabled by bimetallic NiCo hydroxide with layered structure," *Chem. Eng. J.* **2026**, 543, 178093.

[3] Z. Mei, Z. Xu, Z. Tong, et al., "Broadband Visible-Near-Infrared Electrochromism in Fluorene-Bridged Viologens for Energy-Efficient Smart Windows," *Adv. Funct. Mater.* **2026**, 36, e75659.

[4] J. Yang, M. Wang, Z. Meng, et al., "Visible-Infrared Dual-Band Modulation Inorganic Electrochromic Colorful Smart Window," *Nano Lett.* **2026**, 26 3471-3477.

[5] J. Wang, Z. Zhang, G. Wang, et al., "Low-Energy Consumed Switchable Windows with Ultralong Optical Memory via Semiconductor-Enabled Reversible Zinc Electrodeposition," *Adv. Mater.* **2026**, 38, e21622.

[6] B. Xu, W. Wu, Y. Zhang, et al., "Recyclable quasi-solid-state dynamic windows via reversible dual-metal electrodeposition for building energy modulation," *Nat. Commun.* **2025**, 17, 262.

[7] W. J. Lee, P. Mehra, J. R. Thurston, et al., "Robust fast-switching black electrochromic windows based on solution-processed n-doped transparent organic conductor," *Nat. Commun.* **2025**, 17, 575.

[8] C. Wang, S. Xiang, L. Chen, et al., "In Situ Formation of LiF-Rich SEI in Quasi-Solid Electrolytes Enables Long-Life Electrochromic Devices," *Adv. Mater.* **2026**, 38, e16702.

[9] B. Huang, F. Zhao, Y. Xu, et al., "Multicolored Dual-Band Zinc Anode-Based Electrochromic Device with Four-Mode Conversion Based on Phytic Acid-Doped Polyaniline," *Adv. Funct. Mater.* **2025**, 36, e25837.

[10] F. Ping, Y. Zhong, X. Guo, et al., "Unlocking Scalable Manufacturing of Electrochromic Windows: Wadsley-Roth TiNb2O7 Films with Open Shear Channels for Intelligent Photo-Thermal Management," *Adv. Funct. Mater.* **2025**, 36, e27930.

[11] J. Wang, L. Cui, M. Zhang, et al., "Electronic behavior and state changes drive tungsten oxide for ultra-low energy consumption dual-band electrochromic smart windows," *Chem. Eng. J.* **2025**, 525, 170192.

[12] L. Vallan, J. R. Otaegui, D. Ruiz-Molina, et al., "Cost-Effective Paraffin Particles-Based Thermochromic Composites for Smart Windows and Greenhouses", *Small Str.* **2025**, 6, 2500250.

[13] M. Villabona, L. Vallan, D. Ruiz-Molina, et al., "Weather-Resistant Thermoresponsive UV-Curable Smart Window Composites Based on Paraffin Particles ", *ACS Appl. Mater. Interfaces* **2026**, 18, 37058–37068

[14] H. Wang, Y. Lu, J. Wang, et al., "Hydrated ionic polymer for thermochromic smart windows in buildings," *Nat. Commun.* **2025**, 16, 6509.

[15] C. Hong, B. Li, J. Zhang, et al., "Supramolecular Polymer-Based Ionogels Enable Large-Scale Fabrication of Stable Smart Windows with Room-Temperature Closed-Loop Recyclability and Self-Healing Capability," *Adv. Funct. Mater.* **2024**, 34, 2313781.

[16] B. Li, F. Xu, T. Guan, et al., "Self-adhesive self-healing thermochromic ionogels for smart windows with excellent environmental and mechanical stability, solar modulation, and antifogging capabilities," *Adv. Mater.* **2023**, 35, 2211456.

[17] H. Liu, C. Qu, S. Song, et al., "Upper-critical-solution-temperature-type hydrogel smart window with wide temperature regulation for all weather privacy protection and energy

saving," *Chem. Eng. J.* **2025**, 522, 167953.

[18] X. Y. Debbie Soo, Z. M. Png, D. Zhang, et al., "UV-curable flexible thermochromic phase change materials," *Mater. Today Chem.* 2026, 53, 103538.

[19] G. Wang, S. Wang, B. Li, et al., "Submicron Metasurface Reconfigured Multiband Smart Windows," *Adv. Funct. Mater.* **2026**, 36, e76663.

[20] Y. Hu, Q. Huang, H. Chen, et al., "Bidirectional Thermochromic Hydrogel with Wide Response Temperature Regulation for Smart Windows," *ACS Appl. Mater. Interfaces* **2025**, 17, 44975-44988.

[21] Z. Jin, B. Sun, Q. Lu, et al., "Scalable Ionogel-Based Thermochromic Smart Windows: Enhanced Solar Regulation, Weatherability, and Processability," *ACS Appl. Mater. Interfaces* **2026**, 18, 39300–39312.

[22] S. Mo, L. Gao, "All-Season Thermochromic Window With Customizable Optical Switching Temperature and Directional Thermal Radiation," *Adv. Mater.* **2025**, 37, e2504208.

[23] J. Jin, J. Zhang, J. Zhang, et al., "Minute-Level Room-Temperature Switching and Long Cycle Stability of Thermochromic Inorganic Perovskite Smart Windows," *Adv. Mater.* **2025**, 37, e2416146.

[24] H. Peng, W. Lin, Y. Gu, et al., "Electricity-Efficient On-Demand Photothermal Activation for Tunable Thermochromic Windows," *Nano Lett.* **2025**, 25, 5027-5034.

[25] F. Jing, Z. Zhang, L. Zhang, et al., "A Dual-Band Smart Window with Electrical-Thermal Control for Visible and Near-Infrared Light Modulation," *Small* **2025**, 21, e06780.

[26] B. Deng, Y. Zhu, X. Wang, et al., "An ultrafast, energy-efficient electrochromic and thermochromic device for smart windows," *Adv. Mater.* **2023**, 35, 2302685.

[27] H. Peng, R. Fu, H. Wang, et al., "On-demand electro-/thermo-chromic smart windows with self-adaptive sensible heat storage for multimode synergistic building energy conservation," *Appl. Energy* **2026**, 405, 127222.

[28] W. Wang, J. Li, Q. Li, et al., "A dual-mode electrochromic and thermochromic smart window utilizing a polyzwitterionic hydrogel," *J. Colloid Interface Sci.* **2025**, 702, 138834.

[29] F. Sun, H. Wu, M. Li, et al., "Smart window with high solar blocking capability: Integrating electrochromic TiO_2-viologen composite film and thermochromic electrolyte", *Chem. Eng. J.* **2025**, 521, 166975.

[30] L. Wang, H.-F. Nan, H.-R. Wu, et al. "Dual-response electro-thermochromic smart windows enabled by an anti-freezing and highly conductive hydrogel electrolyte", *J. Mater. Chem. A* **2026**, 14, 32100–32111.
